# Coinfection and superinfection in ICU critically ill patients with severe COVID-19 pneumonia and influenza pneumonia: are the pictures different?

**DOI:** 10.3389/fpubh.2023.1195048

**Published:** 2023-08-29

**Authors:** Ziying Chen, Qingyuan Zhan, Linna Huang, Chen Wang

**Affiliations:** ^1^Peking University China-Japan Friendship School of Clinical Medicine, Beijing, China; ^2^National Center for Respiratory Medicine, China-Japan Friendship Hospital, Beijing, China; ^3^State Key Laboratory of Respiratory Health and Multimorbidity, China-Japan Friendship Hospital, Beijing, China; ^4^National Clinical Research Center for Respiratory Diseases, China-Japan Friendship Hospital, Beijing, China; ^5^Institute of Respiratory Medicine, Chinese Academy of Medical Sciences, China-Japan Friendship Hospital, Beijing, China; ^6^Department of Pulmonary and Critical Care Medicine, Center of Respiratory Medicine, China-Japan Friendship Hospital, Beijing, China; ^7^Chinese Academy of Medical Sciences and Peking Union Medical College, Beijing, China

**Keywords:** COVID-19, influenza, coinfection, ICU-acquired superinfection, bacteria

## Abstract

**Background:**

Similar to influenza, coinfections and superinfections are common and might result in poor prognosis. Our study aimed to compare the characteristics and risks of coinfections and superinfections in severe COVID-19 and influenza virus pneumonia.

**Methods:**

The data of patients with COVID-19 and influenza admitted to the intensive care unit (ICU) were retrospectively analyzed. The primary outcome was to describe the prevalence and pathogenic distribution of coinfections/ICU-acquired superinfections in the study population. The secondary outcome was to evaluate the independent risk factors for coinfections/ICU-acquired superinfections at ICU admission. Multivariate analysis of survivors and non-survivors was performed to investigate whether coinfections/ICU-acquired superinfections was an independent prognostic factor.

**Results:**

In the COVID-19 (*n* = 123) and influenza (*n* = 145) cohorts, the incidence of coinfections/ICU-acquired superinfections was 33.3%/43.9 and 35.2%/52.4%, respectively. The most common bacteria identified in coinfection cases were *Enterococcus faecium, Pseudomonas aeruginosa*, *and Acinetobacter baumannii* (COVID-19 cohort) and *A. baumannii, P. aeruginosa*, *and Klebsiella pneumoniae* (influenza cohort). A significant higher proportion of coinfection events was sustained by *Aspergillus* spp. [(22/123, 17.9% in COVID-19) and (18/145, 12.4% in influenza)]. The COVID-19 group had more cases of ICU-acquired *A. baumannii*, *Corynebacterium striatum* and *K. pneumoniae*. *A. baumannii*, *P. aeruginosa*, and *K. pneumoniae* were the three most prevalent pathogens in the influenza cases with ICU-acquired superinfections. Patients with APACHE II ≥18, CD8+ T cells ≤90/μL, and 50 < age ≤ 70 years were more susceptible to coinfections; while those with CD8+ T cells ≤90/μL, CRP ≥120 mg/L, IL-8 ≥ 20 pg./mL, blood glucose ≥10 mmol/L, hypertension, and smoking might had a higher risk of ICU-acquired superinfections in the COVID-19 group. ICU-acquired superinfection, corticosteroid administration for COVID-19 treatment before ICU admission, and SOFA score ≥ 7 were independent prognostic factors in patients with COVID-19.

**Conclusion:**

Patients with COVID-19 or influenza had a high incidence of coinfections and ICU-acquired superinfections. The represent agents of coinfection in ICU patients were different from those in the general ward. These high-risk patients should be closely monitored and empirically treated with effective antibiotics according to the pathogen.

## Introduction

The coronavirus disease 2019 (COVID-19) outbreak has caused a global health crisis and led to a high rate of critical illness (1). Although the Omicron variant might cause milder cases, mortality increased during the Omicron period, even in a highly vaccinated population (2). Influenza virus infection is also a global public health problem that has caused major morbidity and mortality in many countries (3). Both predispose patients to coinfections and superinfections, especially with bacteria, which could promote severe disease and necessitate timely diagnosis (4–6).

Coinfections in patients with COVID-19 seem uncommon, ranging from 0 to 19% (7–12). However, similar to influenza, superinfections are common in COVID-19, which can follow the initial infection phase or occur during recovery (13). Early recognition of patients with a high risk of coinfections/superinfections is important for the early use of antibiotics and in implementing measures to limit the possibility of superinfection, which may, in turn, reduce mortality, especially in the intensive care unit (ICU). It could even reduce antibiotic resistance caused by the unnecessary use of antibiotics. However, as of March 2023, the risk factors and characteristics related to coinfections/superinfections in COVID-19 and influenza cases in the same ICU have not been described.

Therefore, we aimed to describe the prevalence, pathogenic distribution and clinical characteristics of coinfections/superinfections in patients with COVID-19 and influenza in the same ICU. We also explored the predictive factors of coinfections/superinfections, which might help choose the appropriate application time and variety of antibiotics.

## Materials and methods

### Study design and patients

This retrospective observational study included patients with severe influenza virus pneumonia from December 1, 2017, to February 28, 2022, and patients with severe COVID-19 from December 1, 2022, to February 28, 2023, admitted to the respiratory ICU (RICU) of China-Japan Friendship Hospital in China. Patients younger than 18 years of age were excluded.

Demographics, clinical data, and results of microbiological examinations were extracted from the electronic medical record management system. Due to the study’s retrospective nature, the need for informed consent from the patients or their legal guardians was waived. The study was approved by the institutional ethics committees of China-Japan Friendship Hospital.

The primary outcome in our study was a description of the prevalence and pathogenic distribution of coinfections and ICU-acquired superinfections in patients with COVID-19 and influenza. The secondary outcome was an evaluation of the independent risk factors for coinfections/ICU-acquired superinfections at ICU admission. Multivariate analysis of survivors and non-survivors was performed to investigate whether coinfections/ICU-acquired superinfections was an independent prognostic factor.

### Diagnostic criteria

All patients with influenza infection underwent testing using nasopharyngeal swabs or lower respiratory tract (LRT) specimens. Two methods were used for laboratory diagnosis: polymerase chain reaction (PCR) and serological testing (14, 15). Severe influenza virus pneumonia was defined as the presence of influenza infection and severe community-acquired pneumonia (CAP) (16).

SARS-CoV-2 infection was confirmed via viral genome positivity in PCR or antigen, according to the Diagnosis and Treatment of Novel Coronavirus Infection Interim Guidance Report by the National Health Commission of the People’s Republic of China (17). Severe COVID-19 was defined as any one of the following: (1) shortness of breath with respiratory rate ≥ 30 per minute; (2) pulse oxygen saturation ≤ 93% in the resting state; and (3) partial pressure of oxygen/fraction of inspiration oxygen (PaO_2_/FiO_2_) ≤300 mmHg. Critically ill patients satisfied any one of the following criteria: (1) respiratory failure where invasive ventilation is needed, (2) shock, and (3) failure of any other organ and need for ICU care (17).

### Definitions

Coinfection was defined as pathogen detection via diagnostic test at the time of or within the first 48 h of ICU admission. If detection occurred ≥48 h after ICU admission, the infection was defined as an ICU-acquired superinfections. These tests included cultures of the respiratory tract secretions (sputum, bronchoalveolar lavage fluid, and endotracheal aspiration), multiplex respiratory PCRs performed on a nasopharyngeal swab or on respiratory tract secretions, metagenomic next-generation sequencing (mNGS) for respiratory tract secretions, and urinary antigen test for *Streptococcus pneumoniae*. The final diagnosis of causative agents was made according to the clinical physician expert groups combining imaging and clinical symptoms. Tracheobronchitis was defined as follows: The combination of fever (>38°C) with no other recognizable cause, new or increased sputum production, and a positive tracheal aspirate culture without radiographic evidence of pneumonia (18). Pneumonia was defined by the presence of new or progressive radiographic infiltrate associated with two of the following criteria: (1) Fever, temperature above 38°C; (2) leukocyte count above 10 × 10^9^/L or below 4 × 10^9^/L, and (3) purulent endotracheal aspirate (19). Fungal infection was diagnosed according to the taskforce report on the diagnosis and clinical management of COVID-19 associated pulmonary aspergillosis (20) and clinical practice guideline for the management of candidiasis: 2016 Update by the Infectious Diseases Society of America (21).

### Statistical analysis

Continuous variables are presented as mean ± standard deviation or median (interquartile range) and compared using a *t*-test or Mann–Whitney *U* test. Categorical variables were described using percentages and compared using the chi-square or Fisher’s exact tests. All significance tests were two-tailed, and statistical significance was defined as *p* ≤ 0.05. Factors associated with coinfections/ICU-acquired superinfections were evaluated using univariate and multivariate analyzes. The multivariate analysis included all variables (*p* < 0.1) from the univariate analysis and the factors reported to be associated with coinfections/ICU-acquired superinfections. All results were analyzed using SPSS for Windows, version 26 (IBM Corp, Armonk, NY, United States).

## Results

A total of 123 patients with confirmed COVID-19 during the study period were included. The median age of them was 69 (59–78) years, 99/123 (80.5%) of the patients were male. And 87/123 (70.7%) had underlying diseases (60.2% hypertension, 39.8% diabetes, 11.4% chronic heart failure, 22% chronic renal failure, 13.8% chronic lung disease and 40.7% immunocompromised). The Acute Physiology And Chronic Health Evaluation (APACHE) II and Sequential Organ Failure Assessment (SOFA) scores were 17 (12–23) and 6(4–10), respectively. Among them, 78/123 (63.4%) underwent bronchoscopy to obtain bronchoalveolar lavage fluid (BALF) specimens, mNGS of the BALF was performed for 59/123 (48%) patients.

In total, 145 patients with confirmed influenza were identified during three consecutive influenza seasons from December 2017 to February 2022. The median age was 58 (46–69) years, 95/145 (65.5%) were male, and 82 (56.6%) patients had underlying diseases. The APACHE II and SOFA scores were 19 (14–23) and 7 (4–11), respectively (Supplementary Table 3).

### Prevalence and pathogenic distribution of coinfections

**Figure 1:**
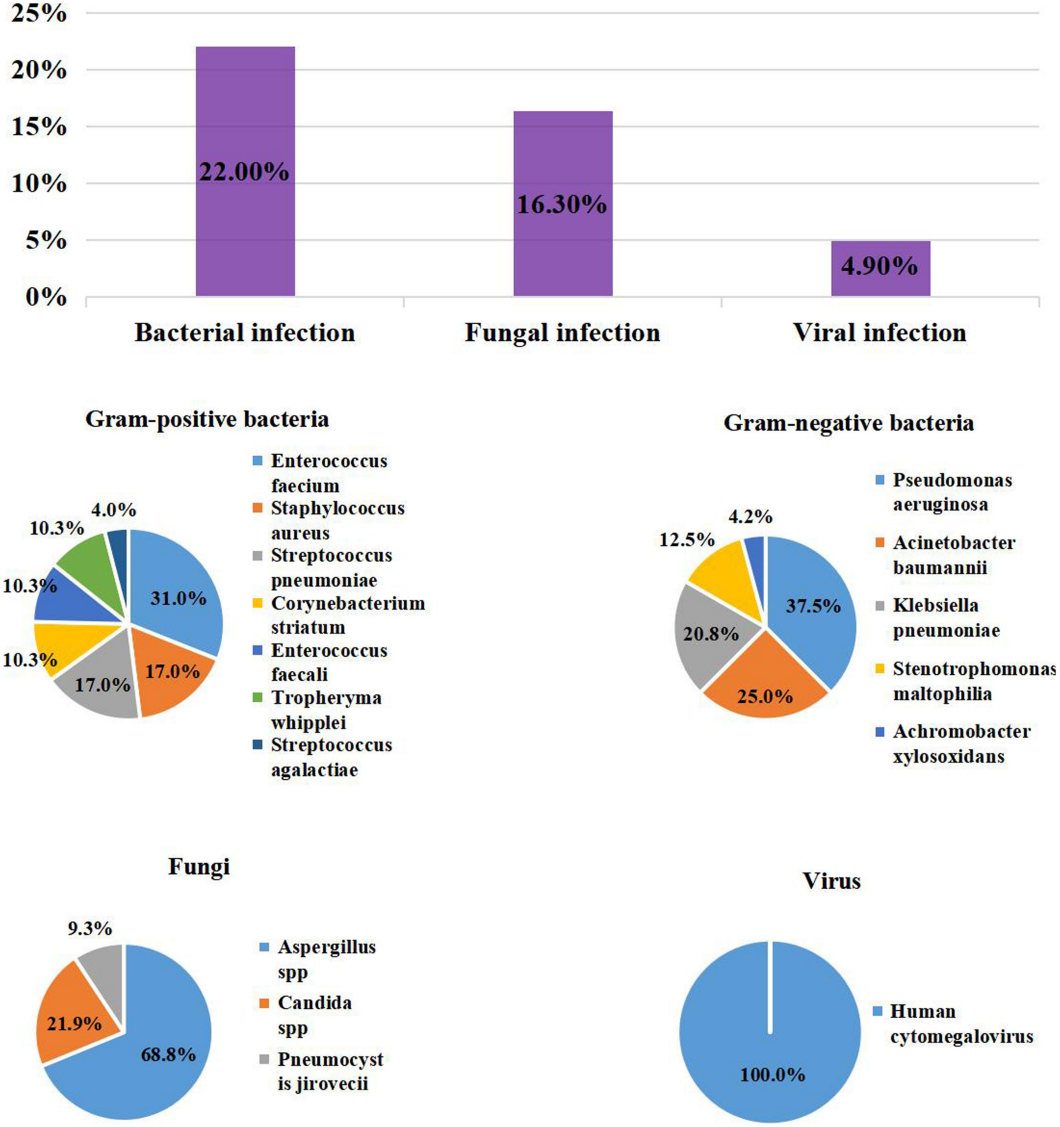
The Prevalence of coinfection and distribution of Pathogens in patients with COVID-19.

Among the 123 patients with COVID-19, 41/123 (33.3%) had coinfections: 27/123 (22%) with bacterial infections, 20/123 (16.3%) with fungal infections, and 6/123 (4.9%) with viral infections. The most common bacteria were *Enterococcus faecium* (9/123, 7.3%), *Pseudomonas aeruginosa* (9/123, 7.3%), *Acinetobacter baumannii* (6/123, 4.9%). The most common gram-positive bacteria were *E. faecium* (9/123, 7.3%), *S. pneumoniae* (5/123, 4.1%) and *Staphylococcus aureus* (5/123, 4.1%), which included 1/5 (20%) case of Methicillin-resistant *S. aureus* (MRSA). The most common gram-negative bacteria were *P. aeruginosa* (9/123, 7.3%)*, A. baumannii* (6/123, 4.9%), and *Klebsiella pneumoniae* (5/123, 4.1%). Of these, multidrug-resistant (MDR) organisms accounted for 16.6% (4/24). *Aspergillus* spp. (22/123, 17.9%) and *Candida spp.* (7/123, 5.7%) were the predominant causes of fungal infection; 6/123 (4.9%) patients with COVID-19 were positive for *Human Cytomegalovirus* (CMV) (Table 1; Figure 1).

**Table 1:** Pathogens in COVID-19 and influenza patients with co-infections and ICU-acquired superinfections.

	Coinfections			ICU-acquired superinfections		
	COVID-19 *N* = 123	Influenza *N* = 145	*p* value	COVID-19 *N* = 123	Influenza *N* = 145	*p*-value
Bacterial infection	27 (22)	29 (20)	0.695	50 (40.7)	69(47.6)	0.255
*Gram-positive*						
*Enterococcus faecium*	9 (7.3)	0	0.001	5 (4.1)	1 (0.7)	0.097
*Staphylococcus aureus*	5 (4.1)	5 (3.4)	1	3 (2.4)	1 (0.7)	0.336
MRSA	1 (20)	2 (40)	1	3 (100)	1 (100)	-
MSSA	0	3 (60)	0.167	0	0	-
*Streptococcus pneumoniae*	5 (4.1)	0	0.019	1 (0.8)	0	0.459
*Streptococcus constellatus*	0	0	-	1 (0.8)	0	0.459
*Corynebacterium striatum*	3 (2.4)	4 (2.8)	1	23 (18.7)	3 (2.1)	<0.001
*Enterococcus faecali*	3 (2.4)	0	0.095	1 (0.8)	0	0.459
*Tropheryma whipplei*	3 (2.4)	0	0.095	0	0	-
*Streptococcus agalactiae*	1 (0.8)	0	0.459	1 (0.8)	0	0.459
*Mycobacterium tuberculosis*	0	1 (0.7)	1	2 (1.6)	1 (0.7)	0.595
*Gram-negtive*						
*Pseudomonas aeruginosa*	9 (7.3)	10 (6.9)	0.894	4 (3.3)	31 (21.4)	<0.001
*Acinetobacter baumannii*	6 (4.9)	14 (9.7)	0.138	27 (22)	41 (28.3)	0.236
*Klebsiella pneumoniae*	5 (4.1)	6 (4.1)	0.976	8 (6.5)	17 (11.7)	0.143
*Escherichia coli*	0	0	0	5 (4.1)	2 (1.4)	0.253
*Stenotrophomonas maltophilia*	3 (2.4)	1 (0.7)	0.336	1 (0.8)	11 (7.6)	0.008
*Achromobacter xylosoxidans*	1 (0.8)	1 (0.7)	1	1 (0.8)	1 (0.7)	1
*Burkholderia cenocepacia*	0	5 (3.4)	0.064	3 (2.4)	17 (11.7)	0.004
*Enterobacter cloacae*	0	0	–	2 (1.6)	4 (2.8)	0.690
*Ralstonia mannitolilytica*	0	2 (1.4)	0.502	2 (1.6)	4 (2.8)	0.690
*Klebsiella aerogenes*	0	0	–	1 (0.8)	0	0.459
*Klebsiella oxytoca*	0	0	–	0	1 (0.7)	1
*Haemophilus influenzae*	0	0	–	1 (0.8)	0	0.459
*Citrobacter koseri*	0	0	–	1 (0.8)	0	0.459
*Elizabethkingia meningoseptica*	0	0	–	1 (0.8)	0	0.459
*Burkholderia polyphagia*	0	0	–	0	3 (2.1)	0.252
*Acinetobacter picotelli*	0	0	–	0	1 (0.7)	1
MDR	4 (16.6)	18 (46.2)	0.017	34 (59.6)	80 (60.2)	0.948
*Fungal infection*	20 (16.3)	20 (13.8)	0.572	15 (12.2)	13 (9.0)	0.389
*Aspergillus spp*	22 (17.9)	18 (12.4)	0.210	16 (13)	6 (4.1)	0.008
*Candida spp*	7 (5.7)	3 (2.1)	0.119	5 (4.1)	8 (5.5)	0.581
*Rhizopus spp*	0	1 (0.7)	1	2 (1.6)	0	0.210
*Pneumocystis jirovecii*	3 (2.4)	0	0.095	0	1 (0.7)	1
*Viral infection*	6 (4.9)	10 (6.9)	0.487	0	19 (13.1)	<0.001
CMV	6 (4.9)	9 (6.2)	0.637	0	17 (11.7)	<0.001
RSV	0	3 (2.1)	0.252	0	2 (1.4)	0.502
*Others*	0	0	–	0	0	–
*Mycoplasma pneumoniae*	0	0	–	0	1 (0.7)	1
*Chlamydia psittaci*	1 (0.8)	0	0.459	0	0	–
*Ureaplasma*	2 (1.6)	0	0.210	0	0	–

COVID-19, coronavirus disease 2019; MRSA, methicillin-resistant *Staphylococcus aureus*; MSSA, methicillin-sensitive *Staphylococcus aureus*; MDR, multiple drug resistant; CMV, human cytomegalovirus; RSV, respiratory syncytial virus.

**Figure 2:**
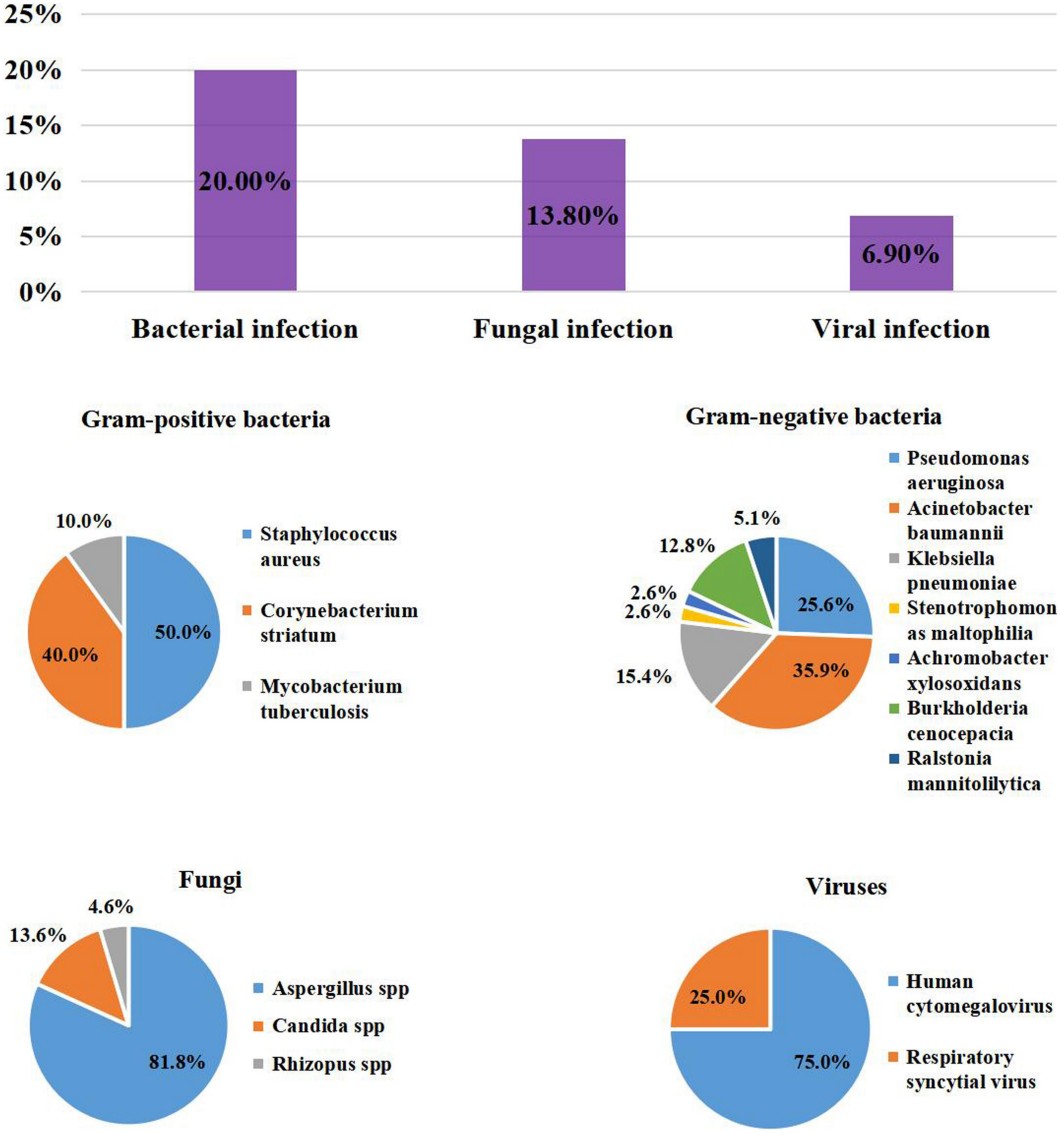
The Prevalence of coinfection and distribution of Pathogens in patients with Inluenza.

Among the patients with influenza, the prevalence of coinfection was 35.2% (51/145), similar to those with severe COVID-19 patients (35.2% vs. 33.3%, *p* = 0.752). Among them, 29/145 (20%) pathogens were bacteria, 20/145 (13.8%) were fungi, and 10/145 (6.9%) were viruses. Gram-positive bacteria mainly included *S. aureus* (5/145, 3.4%), which included 2/5 (40%) MRSA. The main gram-negative bacteria detected were *A. baumannii* (14/145, 9.7%), *P. aeruginosa* (10/145, 6.9%), and *K. pneumoniae* (6/145, 4.1%). The gram-negative bacteria were MDR, being more than those in the COVID-19 group (46.2% vs. 16.6%, *p =* 0.017). For fungi, *Aspergillus* spp. (18/145, 12.4%) was the most frequently reported, followed by *Candida spp.* (3/145, 2.1%) and *Rhizopus spp.* (1/145, 0.7%). *CMV* (9/145, 6.2%) was the most common virus. The prevalence of bacteria, fungi and viruses was also similar to that of the COVID-19 group (Table 1; Figure 2).

### Prevalence and pathogenic distribution of ICU-acquired superinfections

**Figure 3:**
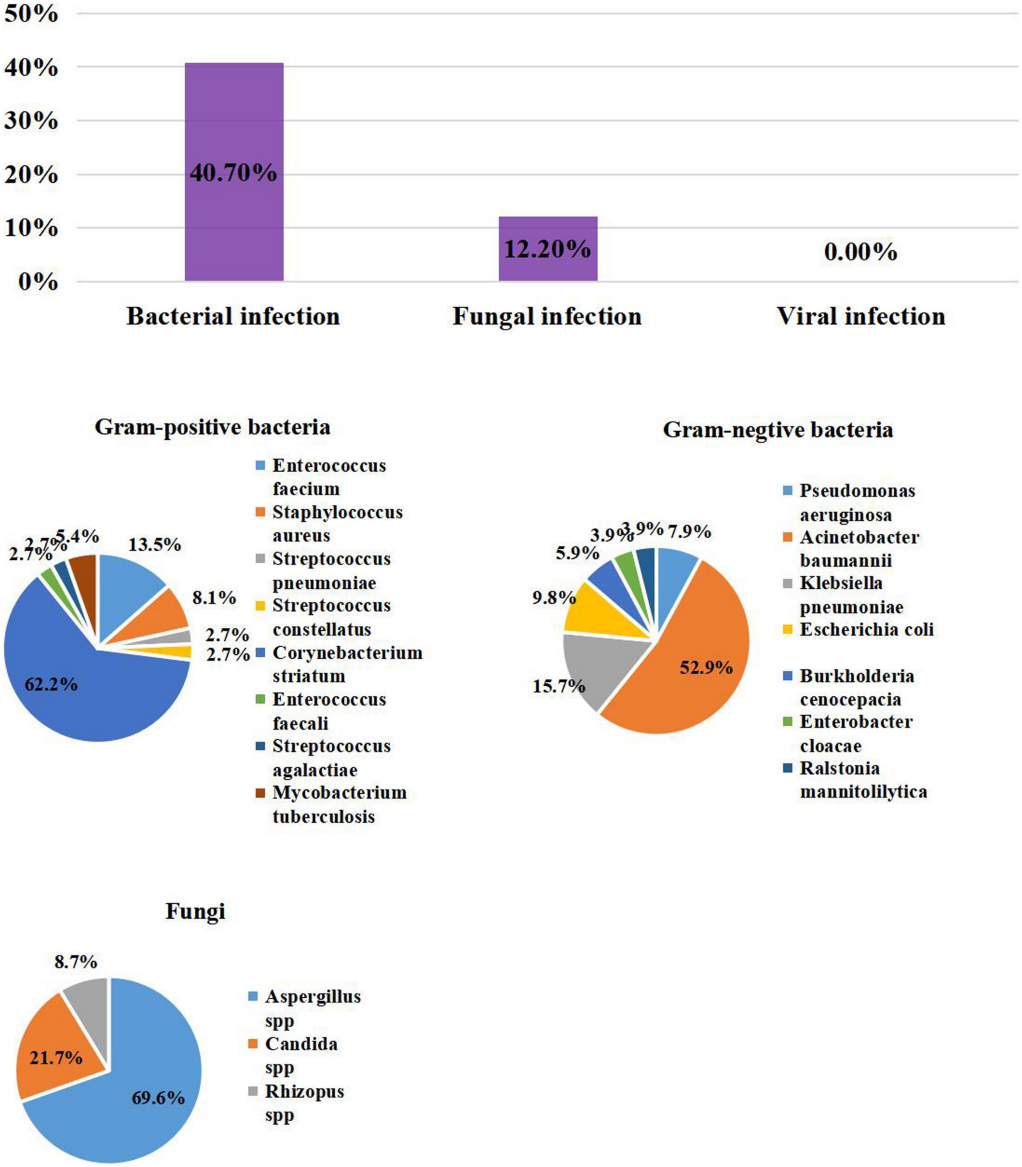
The Prevalence of ICU – acquired infection distribution of Pathogens in patients with COVID-19.

Overall, 54/123 (43.9%) patients with COVID-19 experienced ICU-acquired superinfections: 50/123 (40.7%) and 15/123 (12.2%) had bacterial and fungal infections, respectively. *A. baumannii* (27/123, 22%) was the most common bacteria, followed by *C. striatum* (23/123, 18.7%), *K. pneumoniae* (8/123, 6.5%), *Escherichia coli* (5/123, 4.1%), and *E. faecium* (5/123, 4.1%). Also, 59.6% of the patients had MDR bacteria infections. For fungi, we mainly found *Aspergillus* spp. (16/123, 13%), *Candida spp.* (5/123, 4.1%) and *Rhizopus spp.* (2/123, 1.6%). There was no ICU-acquired virus superinfection in COVID-19 patients (Table 1; Figure 3).

**Figure 4:**
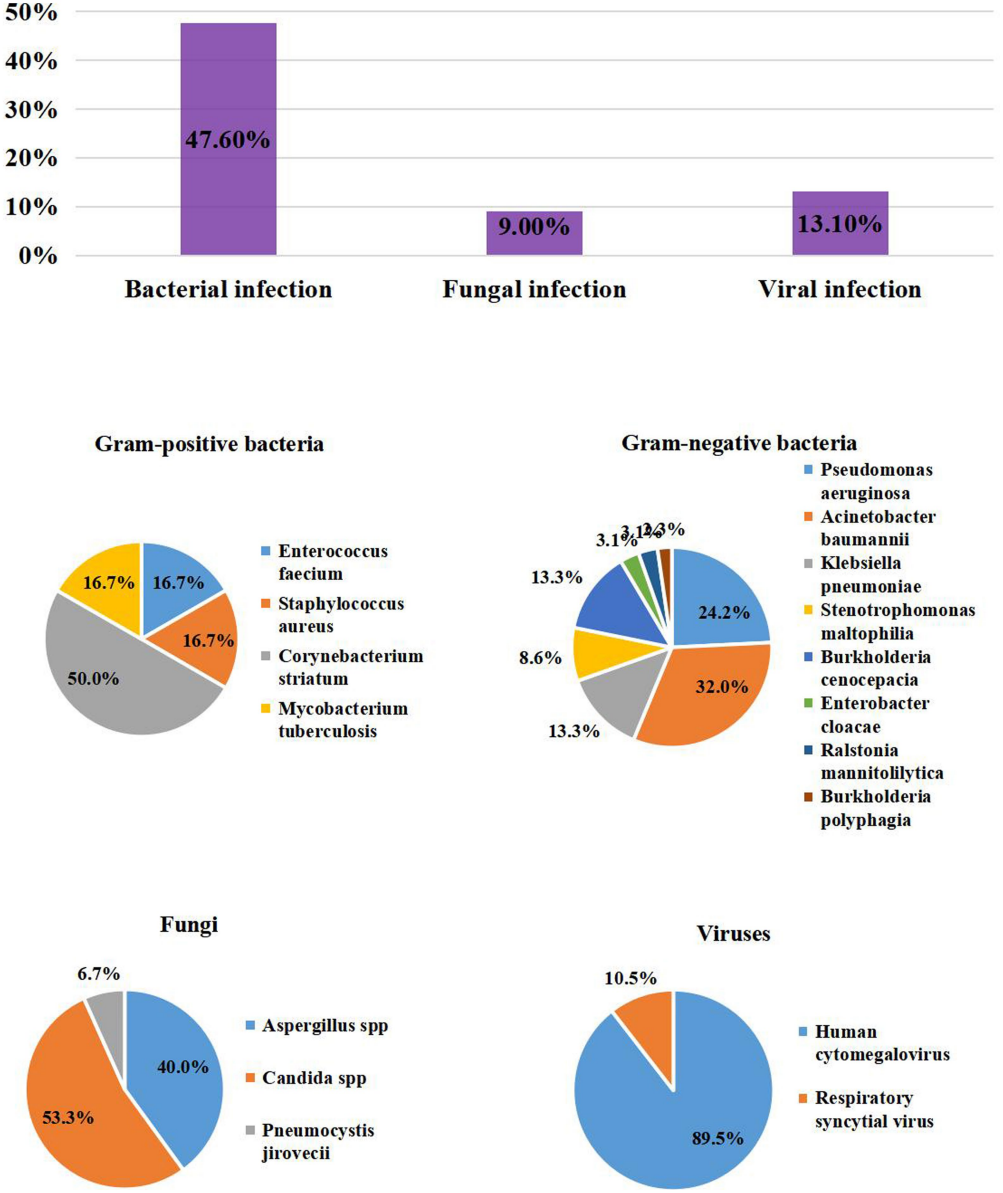
The Prevalence of ICU – acquired infection distribution of Pathogens in patients with Inluenza.

A total of 76/145 (52.4%) patients had ICU-aqcuired superinfections in the influenza group: most were bacteria (69/145, 47.6%), followed by fungi (13/145, 9.0%) and viruses (19/145, 13.1%). The prevalence of bacteria and fungi were similar to that of the COVID-19 group. *A. baumannii* (41/145, 28.3%)*, P. aeruginosa* (31/145, 21.4%)*, K. pneumoniae* (17/145, 11.7%), and *Burkholderia cenocepacia* (17/145, 11.7%) were the most prevalent bacteria. The proportions of *P. aeruginosa, Stenotrophomonas maltophilia*, and *B. cenocepacia* were higher than in the COVID-19 group (21.4% vs. 3.3%, *p* < 0.001; 7.6% vs. 0.8%, *p* = 0.008; 11.7% vs. 2.4%, *p* = 0.004, respectively). However, the influenza group had fewer *Corynebacterium striatum* infections than the COVID-19 group (2.1% vs. 18.7%, *p* < 0.001). In addition，60.2% of the patients had MDR bacteria, similar to the COVID-19 group. The fungal infections included *Candida* spp. (8/145, 5.5%), *Aspergillus spp.* (6/145, 4.1%) and *Pneumocystis jirovecii* (1/145, 0.7%).The influenza group had less *Aspergillus spp.* infection than COVID-19 group (4.1% vs. 13%, *p* = 0.008). ICU-acquired CMV infection accounted for 11.7% (17/145) of causes, which should be of great concern (Table 1; Figure 4).

### Risk factors for coinfections

The univariate analysis of patients with COVID-19 showed no significant differences in age, APACHE II, SOFA, and comorbidities on diagnosis between patients with and without coinfections (Supplementary Table 4). Combining the factors reported in the literature associated with coinfection, we conducted a multivariate analysis. APACHE II ≥18 (OR: 2.309; 95%CI: 1.005–5.304; *p* = 0.049), CD8+ T cells ≤90/μL (OR: 2.466; 95%CI: 1.084–5.612; *p* = 0.031), and 50 < age ≤ 70 years (OR: 2.680; 95%CI: 1.183–6.072; *p* = 0.018) were independent risk factors for coinfection (Table 2).

**Table 2:** Independent risk factors for coinfection of COVID-19 and influenza.

Variable	COVID–19			Influenza		
	*p* value	OR	95% CI	*p* value	OR	95% CI
APACHEII ≥18	0.049	2.309	1.005–5.304	–	–	–
CD8+ T cell ≤90/μL	0.031	2.466	1.183–6.072	–	–	–
50 < Age ≤ 70 years	0.018	2.680	1.183–6.072	–	–	–
BMI ≤23.5 kg/m^2^	–	–	–	0.008	2.722	1.304–5.683
WBC ≥10× 10^9^ /L	–	–	–	0.047	2.102	1.009–4.380

APACHE, acute physiology and chronic health evaluation; BMI, body mass index; WBC, white blood cell.

In the influenza cohort, the time from illness onset to ICU admission was longer in patients with coinfections [13 (7–22) vs. 8 (5.5–11.5), *p* = 0.001]. Patients with coinfections had a lower body mass index (BMI) than those without coinfections [23.38 (21.40–25.90) vs. 25.34 (22.49–28.01), *p* = 0.012]. The white blood cell count and prothrombin time were higher in patients with coinfection (Supplementary Tables 4, 5). After multivariate analysis, BMI ≤23.5 kg/m^2^ (OR: 2.722; 95%CI: 1.304–5.683; *p* = 0.008) and white blood cell (WBC) count ≥10 × 10^9^/L (OR: 2.102; 95% CI: 1.009–4.380; *p* = 0.047) were independent predictive variables for coinfections in patients with influenza (Table 2).

### Risk factors of ICU-acquired superinfections

Patients with COVID-19 who developed ICU-acquired superinfections had higher APACHE II [19.5 (14.25–26) vs. 14 (10.75–21.25), *p* = 0.004] and SOFA [7 (5–10) vs. 4 (2.75–8), *p* = 0.002] scores and higher rates of smoking (48.1% vs. 23.2%, *p* = 0.004), dyspnea (100% vs. 84.1%, *p* = 0.002), and hypertension (72.2% vs. 50.7%, *p* = 0.016; Supplementary Table 1). The levels of neutrophils, C-reactive protein (CRP), procalcitonin, interleukin-6 (IL-6), interleukin-8 (IL-8), and D-dimer were higher and, the level of CD8+ T cells was lower in patients with ICU-acquired superinfections than in those without (Supplementary Tables 6, 7). Multivariate analysis showed that CD8+ T cells ≤90/μL (OR: 6.016; 95%CI: 2.270–15.944; *p* < 0.001), CRP ≥120 mg/L (OR: 4.111; 95%CI: 1.508–11.208; *p* = 0.006), IL-8 ≥ 20 pg./mL (OR: 3.178; 95%CI: 1.233–8.192; *p* = 0.017), blood glucose ≥10 mmol/L (OR: 2.843; 95%CI: 1.101–7.341; *p* = 0.031), hypertension (OR: 2.694; 95%CI: 1.041–6.973; *p* = 0.041), and smoking (OR: 4.599; 95%CI: 1.723–12.275; *p* = 0.002) were independent risk factors for ICU-acquired superinfections in the COVID-19 cohort (Supplementary Table 1).

In patients with influenza, we did not find a significant difference in demographic characteristics between patients with ICU-acquired superinfections and those without. However, patients without ICU-acquired superinfections had a higher rate of fever than those with ICU-acquired superinfections (Supplementary Tables 6, 7). However, in the multivariate analysis, WBC ≥10 × 10^9^/L(OR: 2.419; 95%CI: 1.175–4.983; *p* = 0.017), fever (OR: 0.263; 95%CI: 0.084–0.826; *p* = 0.022), expectoration (OR: 0.328; 95%CI: 0.129–0.835; *p* = 0.019) and dyspnea (OR 4.190; 95%CI: 1.229–14.291; *p* = 0.022) were associated with a higher rate of ICU-acquired superinfections in patients with influenza (Supplementary Table 1).

### Influence of coinfections and ICU-acquired superinfections on treatment and prognosis

In severe COVID-19 pneumonia, the use of antifungal agents (80% vs. 31.6%, *p* < 0.001) was higher in patients with coinfections. Also, the following were significantly higher in patients with coinfections: incidence of acute kidney injury (AKI; 51.4% vs. 20.3%, *p* = 0.001), need for tracheal intubation (80.5% vs. 44.4%, *p* < 0.001), tracheotomy (56.1% vs. 23.8%, *p* < 0.001), extracorporeal membrane oxygenation (20.5% vs. 4.1%, *p* = 0.008), and prone position (61.5% vs. 25.7%, *p* < 0.001). There was a significantly longer duration of intensive positive-pressure ventilation (IPPV) in patients with coinfections [6 (0.25–17) vs. 0 (0–7.5), *p* = 0.003]. However, the treatment and outcomes were similar between patients with and without coinfection in the influenza cohort (Table 3).

**Table 3:** Treatment and outcomes of COVID-19 and influenza patients with coinfection.

	COVID-19 N = 123			Influenza *N* = 145		
	None *N* = 82	Coinfection *N* = 41	*p*	None *N* = 94	Coinfection *N* = 51	*p*
Treatment, *n*%				–	–	–
Paxlovid	42 (54.5)	25 (62.5)	0.409	–	–	–
Corticosteroids	66 (85.7)	37 (92.5)	0.376	–	–	–
Anticoagulation	71 (92.2)	40 (100)	0.093	–	–	–
Bacterial antibiotic	71 (92.2)	40 (100)	0.093	–	–	–
Antifungal antibiotic	24 (31.6)	32 (80)	<0.001	–	–	–
Barotrauma	7 (11.5)	6 (16.2)	0.548	4 (4.3)	4 (7.8)	0.452
Acute kidney injury, n%	15 (20.3)	18 (51.4)	0.001	38 (40.4)	24 (47.1)	0.441
Cardiovascular failure, n%	27 (36.5)	15 (42.9)	0.523	21 (22.3)	14 (27.5)	0.492
Acute liver injury, n%	10 (13.5)	3 (8.6)	0.543	14 (14.9)	5 (9.8)	0.386
Hospital acquired pneumonia, n%	31 (37.8)	23 (56.1)	0.054	48 (51.1)	28 (54.9)	0.659
Urinary infection, n%	4 (5.3)	0	0.299	1 (1.1)	1 (2)	1
Abdominal infection, n%	2 (2.7)	1 (2.8)	1	0	2 (3.9)	0.122
Bloodstream infection, n%	3 (4)	4 (11)	0.211	2 (2.1)	2 (3.9)	0.613
Deep venous thrombosis, n%	19 (25.7)	9 (25.7)	0.997	–	–	–
Pulmonary embolism, n%	1 (1.4)	1 (2.8)	0.549	–	–	–
Gastrointestinal bleeding, n%	15 (20.3)	11 (31.4)	0.202	–	–	–
The need of CRRT, n%	18 (26.1)	13 (34.2)	0.375	30 (31.9)	20 (39.2)	0.377
The need of tracheal intubation, n%	36 (44.4)	33 (80.5)	<0.001	66 (71)	39 (76.5)	0.477
The need of tracheotomy, n%	19 (23.8)	23 (56.1)	<0.001	–	–	–
The need of ECMO, n%	3 (4.1)	8 (20.5)	0.008	25 (26.6)	11 (21.6)	0.503
The need of prone position, n%	19 (25.7)	24 (61.5)	<0.001	–	–	–
The need of recruitment, n%	4 (5.6)	3 (7.9)	0.691	–	–	–
The length of IPPV, days	0 (0–7.5)	6 (0.25–17)	0.003	11 (6–23.75)	7 (4–16.25)	0.126
ICU length of stay, days	8 (3–14)	9 (6–20)	0.121	11 (6–21)	9 (6–19.75)	0.497
Hospital length of stay, days	12 (7–18)	16 (7–25)	0.287	20 (10–59)	17 (10–28)	0.877
Hospital survival, n%	37 (45.1)	12 (9.8)	0.152	54 (57.4)	54 (45.1)	0.325

CRRT, continuous renal replacement therapy; ECMO, extracorporeal membrane oxygenation; IPPV, intensive positive–pressure ventilation; ICU, intensive care unit.

The need of tracheal intubation (90.7% vs. 29.4%, *p* < 0.001 and 89.5% vs. 54.4%, *p* < 0.001), the length of IPPV (10.5 days vs. 0 days, *p* < 0.001 and 14 days vs. 5 days, *p* < 0.001), and length of ICU stay (14 days vs. 5.5 days, *p* < 0.001 and 18 days vs. 7 days, *p* < 0.001) were higher in patients with ICU-acquired superinfections in both the COVID-19 and influenza groups. In the COVID-19 group, patients with ICU-acquired superinfections, had higher rates of AKI (50% vs. 14.8%, *p* < 0.001), cardiovascular failure (52.1% vs. 27.9%, *p* = 0.010), gastrointestinal bleeding (34% vs. 16.1%, *p* = 0.030), CRRT (44.9% vs. 15.5%, *p* = 0.001), tracheotomy (66.7% vs. 9%, *p* < 0.001), prone position (62% vs. 19%, *p* < 0.001) and recruitment (11.8% vs. 1.7%, *p* = 0.048; (Table 4)).

**Table 4:** Treatment and outcomes of COVID-19 and influenza patients with ICU-acquired superinfections.

	COVID-19 *N* = 123			Influenza *N* = 145		
	None *N* = 69	ICU-acquired superinfection *N* = 54	*p*	None *N* = 69	ICU-acquired superinfection *N* = 76	*p*
Treatment, *n*%						
Paxlovid	32 (49.2)	35 (67.3)	0.05	–	–	–
Corticosteroids	56 (86.2)	47 (90.4)	0.484	–	–	–
Anticoagulation	62 (95.4)	49 (94.2)	1	–	–	–
Bacterial antibiotic	59 (90.8)	52 (100)	0.033	–	–	–
Antifungal antibiotic	19 (29.7)	37 (71.2)	<0.001	–	–	–
Barotrauma	5 (9.4)	8 (17.8)	0.225	2 (2.9)	6 (7.9)	0.280
Acute kidney injury, n%	9 (14.8)	24 (50)	<0.001	24 (34.8)	38 (50)	0.064
Cardiovascular failure, n%	17 (27.9)	25 (52.1)	0.010	16 (23.2)	23 (30.3)	0.337
Acute liver injury, n%	7 (11.5)	6 (12.5)	0.870	6 (8.7)	13 (17.1)	0.148
Hospital acquired pneumonia, n%	0	54 (100)	<0.001	0	76 (100)	<0.001
Urinary infection, n%	2 (3.2)	2 (3.9)	0.614	0	2 (2.6)	0.498
Abdominal infection, n%	1 (1.6)	2 (4.1)	0.582	0	2 (2.6)	0.498
Bloodstream infection, n%	2 (3.2)	5 (10.2)	0.237	1 (1.4)	3 (3.9)	0.622
Deep venous thrombosis, n%	18 (29)	10 (21.3)	0.359	–	–	–
Pulmonary embolism, n%	2 (3.2)	0	0.504	–	–	–
Gastrointestinal bleeding, n%	10 (16.1)	16 (34)	0.030	–	–	–
The need of CRRT, n%	9 (15.5)	22 (44.9)	0.001	19 (27.5)	31 (40.8)	0.094
The need of tracheal intubation, n%	20 (29.4)	49 (90.7)	<0.001	37 (54.4)	68 (89.5)	<0.001
The need of tracheotomy, n%	6 (9)	36 (66.7)	<0.001	–	–	–
The need of ECMO, n%	3 (4.9)	8 (15.4)	0.061	10 (14.5)	26 (34.2)	0.006
The need of prone position, n%	12 (19)	31 (62)	<0.001	–	–	–
The need of recruitment, n%	1 (1.7)	6 (11.8)	0.048	–	–	–
The length of IPPV, days	0 (0–1)	10.5 (6–15)	<0.001	5 (3–9.5)	14 (7–32)	<0.001
ICU length of stay, days	5.5 (3–10)	14 (8–20.8)	<0.001	7 (4–10.5)	18 (10–35.5)	<0.001
Hospital length of stay, days	11 (6.5–19.5)	15 (8–22)	0.104	11.5 (5.8–16.3)	28.5 (15–57.5)	<0.001
Hospital survival, n%	39 (56.5)	10 (18.5)	<0.001	36 (55.2)	41 (53.9)	0.455

CRRT, continuous renal replacement therapy; ECMO, extracorporeal membrane oxygenation; IPPV, intensive positive–pressure ventilation; ICU, intensive care unit.

In the COVID-19 group, the hospital survival rate was lower in patients with ICU-acquired superinfections (18.5% vs. 56.5%, *p* < 0.001). By comparing survivors and non-survivors, we found that ICU-acquired superinfection (OR: 3.677; 95%CI: 1.518–8.906; *p* = 0.004), corticosteroid administration for COVID-19 treatment before ICU admission (OR: 0.317; 95%CI: 0.125–0.805; *p* = 0.016), and SOFA score ≥ 7 (OR: 6.710; 95%CI: 2.536–17.754; *p* < 0.001) were independent prognostic factors (Supplementary Table 2). However, the hospital survival rate was similar regardless of ICU-acquired superinfection in patients with severe influenza virus pneumonia.

## Discussion

In this study, the prevalence of respiratory coinfections/ICU-acquired superinfections in the COVID-19 and influenza cohorts were 33.3%/43.9 and 35.2%/52.4%, respectively. Bacteria were isolated more frequently not only in coinfection but also in ICU-acquired superinfections cases. The most common bacteria identified in coinfection cases were *P. aeruginosa*, *E. faecium, and A. baumannii* in patients with COVID-19 and *P. aeruginosa*, *A. baumannii, and K. pneumoniae* in patients with influenza. Besides, a significant higher proportion of coinfection events was sustained by *Aspergillus* spp. The COVID-19 group had more cases of ICU-acquired *A. baumannii, C. striatum* and *K. pneumoniae*. *P. aeruginosa*, *A. baumannii*, and *K. pneumoniae* were the three most prevalent pathogens in the influenza cases with ICU-acquired superinfections. In addition, ICU-acquired superinfection was an independent prognostic factor in the COVID-19 group.

Our study demonstrated a higher proportion of patients with coinfections in the COVID-19 group (33.3%). Previous studies and reviews have reported variable coinfection rates, ranging from 3.5 to 14%, focusing on patients from general wards with bacterial infections (7, 8, 11, 12, 22–24). Only a few studies have reported coinfection data from COVID-19 cohort in the ICU (26.9–28%) (11, 25, 26). The coinfection rate in our ICU was higher than that in others. In addition, the main pathogens were gram-negative bacilli and *Aspergillus spp.*, which was not in accordance with previous studies that reported that *S. aureus* and other common community-acquired bacteria were prevalent in coinfections (8, 11, 26). In our influenza cohort, the prevalence of coinfections was similar to most of previous study (4, 27), but higher than others (28, 29).

Meanwhile, unlike other studies, which reported *S. aureus*, *S. pneumoniae* and *Hemophilus influenza* were the most commonly isolated co-infectious agents (4, 5, 27, 29), we found typical pathogens of nosocomial infections such as *A. baumannii* and *P. aeruginosa*. Some factors may explain this phenomenon. First, in our study, the time from onset to ICU admission was longer than in other studies (9 vs. 3–5.6 days). Only, 17.2% of the patients in our study were admitted directly from the emergency or outpatient department. The rest were hospitalized in general wards and other ICUs before being transferred to the ICU in our study. Second, we supposed that it was due to the inclusion bias in different studies but not the actual situation. The diagnostic criteria for coinfection were not standardized, which confounded pathogenicity and colonization. Most of studies that assumed coinfection as the pathogen were detected. In addition, the time of diagnosis of coinfection was not uniform. Many studies included secondary infection or mixed coinfection and secondary infection. Obviously, the proportion of coinfection with *Aspergillus* spp. was quite high in our study. Awareness of the possibility of fungal coinfection in COVID-19 is essential to initiate empirical antifungal therapy and fungal infection test as early as possible, which assisted in preventing severe illness and death from coinfection.

In the COVID-19 cohort, patients aged 50–70 years had the highest prevalence of coinfection (OR: 2.680), which was in agreement with studies that reported that older adult patients tended to have coinfection (30, 31). Hughes et al. (8) indicated that the age group of 55–81 years were predisposed to coinfection, which concorded with our study (50–70 years). APACHE II ≥18 and CD8+ T cell ≤90/μL were also independent risk factors for coinfection. These findings might imply that critical conditions due to disease and declined immune ability due to aging are the causes of coinfection in the older adult (32). In addition, BMI ≤23.5 kg/m^2^ and WBC ≥10 × 10^9^/L were predictive factors of influenza coinfection. A higher WBC count was a manifestation of coinfection. A large prospective study conducted by Langouche et al. reported that critical illness evokes adipose tissue accumulation of alternatively active M2 macrophages, which have local anti-inflammatory functions (33). Patients with a lower BMI may have weak anti-inflammatory abilities. Our study showed that patients with high-risk factors could be treated empirically with antibiotics after ICU admission. Antibiotics should cover both gram-positive and gram-negative bacteria. It should also be noted that patients with COVID-19 may have coinfection with fungi, and these patients should be promptly treated with antifungal therapy.

The pathogenesis of influenza coinfection has been elaborated. Influenza virus contributes to respiratory epithelial cell damage, bacterial mucociliary clearance dysfunction, and immune response dampening, enabling increased bacterial adherence and invasion (34, 35). As for COVID-19, the mechanism of the pathogenesis of coinfection remains indistinct, and we lack evidence to support the bacteria-virus association (7).

The incidence of ICU-acquired superinfections was similar in the COVID-19 and influenza cohort (43.9% vs. 53.4%). In both cohorts, gram-negative bacilli were responsible for most ICU-acquired superinfections. *A. baumannii*, *K. pneumoniae,* and *P. aeruginosa* were the most commonly identified bacteria. However, the COVID-19 group had significantly higher rates of *C. striatum* and lower rates of *P. aeruginosa*, *S. maltophilia*, *B. cenocepacia,* and *CMV* than the influenza cohort. Possibly due to different periods, the distribution of nosocomial bacteria in the ICU was different. *CMV* infection is generally asymptomatic and usually presents as a latent infection in healthy individuals. Immune abnormalities caused by influenza may promote *CMV* expression (36).

Further, the incidence of MDR bacteria was similar between COVID-19 and influenza. In the COVID-19 group, 2 patients had ICU-acquired superinfections with *Aspergillus* spp. and *Rhizopus spp*. They all experienced kidney transplantation and took immunosuppressants for long periods, which accounted for the immune disorder. In a multicenter cohort study conducted by Rouze et al. (37), the incidence of ventilator-associated lower respiratory tract infection was significantly higher in the COVID-19 group (50.5%) than in the influenza group (30.3%). However, we did not reach this conclusion. The shorter duration of IPPV for COVID-19 in our study may have contributed to this difference. Like VAP in other diseases (38), ICU-acquired superinfection was associated with longer IPPV time, ICU stay and hospital stay. Another key finding from our study was that ICU-acquired superinfection was associated with a reduced survival rate in patients with COVID-19. This results agrees with those of other studies that have shown a negative association between secondary infection and an increased risk of death (39, 40).

This study identified many predictive factors for ICU-acquired superinfection of COVID-19. The decrease in CD8+ T cells and the increase of IL-8 levels indicated the cytokine storm activation and subsequent immunosuppression (41). Immune response dysfunction may be associated with a higher risk of ICU-acquired superinfection. Moreover, high blood glucose levels, hypertension, and smoking have all reported to be related to secondary infection (42–44). Based on the above results, early identification of high-risk patients for ICU-acquired superinfection and active examination for pathogens will facilitate timely and appropriate antibiotics, which is beneficial to prognosis.

To our knowledge, this is the first study to focus on the coinfections/ICU-acquired superinfections of COVID-19 versus influenza ICU patients in Asia. We clearly defined the time of sample positivity time from the date of ICU admission (<48 h vs. ≥ 48 h). However, this study had several limitations. First, this was a single-center retrospective study, and the study population was relatively small; therefore, possible selection and report biases exist, and it is difficult to generalize the results to other centers. Second, ICU management, ICU isolation measures, sampling methods, and infection diagnostic techniques differed between the COVID-19 and influenza pandemics. In addition, we distinguished infection and colonization using clinical judgment rather than bacterial count, which may have affected the detection rate. Most importantly, some patients were exposed to antibiotics and stayed in general wards or other ICUs before our ICU admission. This could have impacted pathogenic microorganism detection and potentially underestimated or overestimated the real coinfection rate.

## Conclusion

Coinfections and ICU-acquired superinfections were frequent not only in COVID-19 patients but also in influenza patients admitted to the ICU. The represent agents of coinfections in ICU patients were different from those in the general ward. Our study provides evidence supporting close monitoring and empirical choice of antibiotics according to the pathogen for COVID-19 and influenza cases at risk of coinfections/ICU-acquired superinfections in the ICU. Apart from the limited study population, ICU management, ICU isolation measures, sampling methods, and infection diagnostic techniques may have impact on our conclusion. A large-scale and well-designed RCT is needed in the future.

## Data availability statement

The original contributions presented in the study are included in the article/Supplementary material, further inquiries can be directed to the corresponding authors.

## Ethics statement

The studies involving humans were approved by China-Japan Friendship Hospital. The studies were conducted in accordance with the local legislation and institutional requirements. The ethics committee/institutional review board waived the requirement of written informed consent for participation from the participants or the participants’ legal guardians/next of kin due to the study’s retrospective nature.

## Author contributions

LH and QZ took full responsibility for the integrity of the submission and publication and were involved in the study design. QZ, LH, and ZC participated in the design of the study and coordination. ZC involved in data collection, had full access to all of the data in the study, took responsibility for the integrity of the data, and were responsible for data verification. ZC and LH took the responsibility for statistical analysis and drafted the manuscript. QZ provided crucial revision for important intellectual content. All authors made substantial contributions to the conception and design of the study or to the data acquisition, analysis or interpretation, reviewed and approved the final manuscript, and significantly contributed to this study.

## Funding

The funding source of the study [CAMS Innovation Fund for Medical Sciences (CIFMS) 2022-I2M-JB-016] is an academic non-profit organization that played no role in the study design, data collection and analysis, decision to publish, or preparation of the manuscript.

## Conflict of interest

The authors declare that the research was conducted in the absence of any commercial or financial relationships that could be construed as a potential conflict of interest.

## Publisher’s note

All claims expressed in this article are solely those of the authors and do not necessarily represent those of their affiliated organizations, or those of the publisher, the editors and the reviewers. Any product that may be evaluated in this article, or claim that may be made by its manufacturer, is not guaranteed or endorsed by the publisher.
