# Supplementary material for: Coinfection and superinfection in ICU critically ill patients with severe COVID-19 pneumonia and influenza pneumonia: are the pictures different?
Source: Front Public Health. 2023 Aug 29;11:1195048. doi: 10.3389/fpubh.2023.1195048 (PMC10497876; doi:10.3389/fpubh.2023.1195048)
Supplement: Supplementary file 1 [file Table_1.DOCX]

Supplementary, Table 1. Independent risk factors for ICU-acquired superinfection of COVID-19 and influenza

| Variable | COVID-19 | | |  | Influenza | | | |
| --- | --- | --- | --- | --- | --- | --- | --- | --- |
|  | P value | OR | 95% CI |  | P value | | OR | 95% CI |
| CD8+ T cell ≤90/μL | <0.001 | 6.016 | 2.270–15.944 |  | – | – | | – |
| CRP ≥120mg/L | 0.006 | 4.111 | 1.508–11.208 |  | – | – | | – |
| IL–8 ≥20pg/mL | 0.017 | 3.178 | 1.233–8.192 |  | – | – | | – |
| Glu ≥10mmol/L | 0.031 | 2.843 | 1.101–7.341 |  | – | – | | – |
| Hypertension | 0.041 | 2.694 | 1.041–6.973 |  | – | – | | – |
| Smoking | 0.002 | 4.599 | 1.723–12.275 |  | – | – | | – |
| WBC ≥10× 10^9^ /L | – | – | – |  | 0.017 | 2.419 | | 1.175–4.983 |
| Fever | – | – | – |  | 0.022 | 0.263 | | 0.084–0.826 |
| Expectoration | – | – | – |  | 0.019 | 0.328 | | 0.129–0.835 |
| Dyspnea | – | – | – |  | 0.022 | 4.190 | | 1.229–14.291 |

ICU, intensive care unit; CRP, C-reaction protein; IL-8, interleukin-8; Glu, glucose; WBC, white blood cell

Supplementary, Table 2. Independent risk factors for ICU mortality of COVID-19 and influenza

| Variable | COVID-19 | | |  | Influenza | | |
| --- | --- | --- | --- | --- | --- | --- | --- |
|  | P value | OR | 95% CI |  | P value | OR | 95% CI |
| Receiving corticosteroid treatment for COVID-19 before ICU admission | 0.016 | 0.317 | 0.125–0.805 |  | – | – | – |
| ICU-acquired superinfection | 0.004 | 3.677 | 1.518–8.906 |  | – | – | – |
| SOFA≥7 | <0.001 | 6.710 | 2.536–17.754 |  | – | – | – |
| Chronic lung diseases | – | – | – |  | 0.020 | 2.875 | 1.177–7.022 |
| Receiving corticosteroid before ICU | – | – | – |  | 0.014 | 2.857 | 1.235–6.608 |
| PaO_2_/FiO_2_ ≤130mmHg | – | – | – |  | <0.001 | 4.976 | 2.246–11.023 |
| CD4+ T cell ≤200/μl | – | – | – |  | 0.008 | 2.885 | 1.314–6.334 |

ICU, intensive care unit; SOFA, sequential organ failure assessment

Supplementary, Table 3. Demographic and clinical characteristics of COVID-19 and influenza patients

|  | COVID-19  n=123 | Influenza  n=145 | P value |
| --- | --- | --- | --- |
| Age (y) | 69(59–78) | 58(46–69) | <0.001 |
| Male (n%) | 99(80.5) | 95(65.5) | 0.006 |
| APACHE II | 17(12–23) | 19(14–23) | 0.332 |
| SOFA | 6(4–10) | 7(4–11) | 0.053 |
| BMI (kg/m^2^) | 24.06(21.25–26.34) | 24.6(22.05–27.68) | 0.179 |
| Smoking (n%) | 42(34.1) | 57(39.3) | 0.383 |
| Alcoholic (n%) | 28(22.8) | 41(28.9) | 0.258 |
| Underlying diseases (n%) | 87(70.7) | 82(56.6) | 0.017 |
| Diabetes | 49(39.8) | 50(34.5) | 0.265 |
| Chronic heart failure | 14(11.4) | 16(11.1) | 0.944 |
| Chronic renal failure | 27(22) | 13(9.0) | 0.003 |
| Chronic hepatic insufficiency | 2(1.6) | 3(2.1) | 1 |
| Chronic lung diseases | 17(13.8) | 33(22.8) | 0.061 |
| Connective tissue diseases | 13(10.6) | 11(7.6) | 0.520 |
| Solid malignant tumor | 9(7.3) | 10(6.9) | 0.906 |
| Hematological malignant tumor | 6(4.9) | 3(2.1) | 0.309 |
| Receiving corticosteroid therapy | 35(28.5) | 42(29.2) | 0.898 |
| Receiving immunosuppressive drugs | 39(31.7) | 10(6.9) | <0.001 |
| Immunocompromised | 50(40.7) | 53(36.6) | 0.492 |
| Time from illness onset to ICU admission | 13(9–19) | 9(6–15) | <0.001 |
| Location before ICU admission | | | |
| Emergency department. | 61(49.6) | 25(17.2) | <0.001 |
| Hospital ward | 52(42.3) | 103(71.5) | <0.001 |
| Another ICU | 10(8.1) | 16(11.1) | 0.423 |
| Tracheal intubation, n% | 68(56.2) | 105(72.4) | 0.006 |
| The length of IPPV, days | 1(0–10) | 6(0–16.5) | <0.001 |
| Coinfection | 41(33.3) | 51(35.2) | 0.752 |
| ICU-acquired superinfection | 54(43.9) | 76(52.4) | 0.165 |

APACHE, acute physiology and chronic health evaluation; SOFA, sequential organ failure assessment; BMI, body mass index; IPPV, intensive positive–pressure ventilation; ICU, intensive care unit

Immunocompromised patients include following condition: primary immune deficiency diseases; active malignancy or malignancy within 1 y of CAP, excluding patients with localized skin cancers or early–stage cancers (eg, stage 1 lung cancer); receiving cancer chemotherapy; HIV infection with a CD4+ T lymphocyte count < 200 cells/mL or percentage < 14%; solid organ transplantation; hematopoietic stem cell transplantation; receiving corticosteroid therapy with a dose≥20 mg prednisone or equivalent daily for ≥14 d or a cumulative dose > 600 mg of prednisone; receiving biological immune modulators; receiving disease–modifying antirheumatic drugs or other immunosuppressive drugs (eg, cyclosporin, cyclophosphamide, hydroxychloroquine, methotrexate).

Supplementary, Table 4. Demographic and clinical Characteristics of COVID-19 and influenza patients with coinfection

|  | COVID-19 N=123 | | |  | Influenza N=145 | | |
| --- | --- | --- | --- | --- | --- | --- | --- |
|  | None  N=82 | Coinfection  N=41 | p |  | None  N=94 | Coinfection  N=51 | p |
| Age (y) | 69(59–80) | 64(59–71) | 0.072 |  | 60(47–69) | 52(44–67) | 0.212 |
| Male (n%) | 64(78) | 35(85.4) | 0.334 |  | 58(61.7) | 37(72.5) | 0.189 |
| APECHE II | 15(11–22) | 18(14–25) | 0.06 |  | 18(13–23) | 19(14–24) | 0.178 |
| SOFA | 5.5(3–10) | 7(4.25–9.75) | 0.177 |  | 7(4–11) | 9(4–12) | 0.255 |
| BMI (kg/m^2^) | 24.04(20.70–26.72) | 24.06(21.97–26.08) | 0.801 |  | 25.34(22.49–28.01) | 23.38(21.40–25.90) | 0.012 |
| Smoking (n%) | 26(31.7) | 16(37) | 0.420 |  | 36(38.3) | 21(41.2) | 0.735 |
| Alcoholic (n%) | 17(20.6) | 11(26.8) | 0.447 |  | 27(29) | 14(27.5) | 0.841 |
| Vaccinated (n%) | | | | | | | |
| 0 | 16(19.5) | 12(29.3) |  |  | – | – | – |
| 1 |  |  |  |  | – | – | – |
| 2 | 3(3.7) | 0 |  |  | – | – | – |
| 3 | 9(11) | 5(12.2) |  |  | – | – | – |
| Clinical manifestation (n%) | | | | | | | |
| Fever | 73(89) | 40(97.6) | 0.162 |  | 78(83.9) | 44(86.3) | 0.701 |
| Cough | 65(79.3) | 33(80.5) | 0.874 |  | 83(88.3) | 47(92.2) | 0.466 |
| Expectoration | 59(72) | 27(65.9) | 0.487 |  | 74(78.7) | 40(78.4) | 0.967 |
| Running nose | 3(3.7) | 2(4.9) | 1 |  | – | – | – |
| Rhinobyon | 1(1.2) | 1(2.4) | 1 |  | – | – | – |
| Pharyngalgia | 11(13.4) | 6(14.6) | 0.853 |  | – | – | – |
| Dyspnea | 73(89) | 39(95.1) | 0.333 |  | 84(89.4) | 45(88.2) | 0.836 |
| Chest stuffiness | 26(31.7) | 8(19.5) | 0.154 |  | – | – | – |
| Chest pain | 1(1.2) | 1(2.4) | 1 |  | 8(8.5) | 5(9.8) | 0.770 |
| Hemoptysis | 1(1.2) | 0 | 1 |  | 9(9.6) | 12(23.5) | 0.023 |
| Blood–stained sputum | 7(8.5) | 6(14.6) | 0.355 |  | – | – | – |
| Myalgias | 10(12.2) | 7(17.1) | 0.460 |  | – | – | – |
| Headache | 8(9.8) | 1(2.4) | 0.269 |  | – | – | – |
| Fatigue | 22(66.7) | 13(31.7) | 0.572 |  | – | – | – |
| Underlying disease (n%) | | | |  |  |  |  |
| Hypertension | 46(56.1) | 28(68.3) | 0.193 |  | – | – | – |
| Diabetes | 29(35.4) | 20(48.8) | 0.152 |  | 36(38.3) | 13(25.5) | 0.119 |
| Chronic heart failure | 11(13.4) | 3(7.3) | 0.382 |  | 12(12.8) | 4(7.8) | 0.366 |
| Chronic renal failure | 16(19.5) | 11(26.8) | 0.355 |  | 8(8.6) | 5(9.8) | 0.772 |
| Chronic hepatic insufficiency | 0 | 2(4.9) | 0.109 |  | 2(2.1) | 1(2.0) | 1 |
| Chronic lung diseases | 13(15.9) | 4(9.8) | 0.356 |  | 17(18.1) | 16(31.4) | 0.068 |
| Connective tissue diseases | 12(14.6) | 1(2.4) | 0.058 |  | 6(6.4) | 5(9.8) | 0.518 |
| Solid malignant tumor | 5(6.1) | 3(7.3) | 1 |  | 8(8.5) | 2(3.9) | 0.495 |
| Hematological malignant tumor | 4(4.9) | 1(2.4) | 0.664 |  | 3(3.2) | 0 | 0.552 |
| Immunocompromised | 32(39) | 16(39) | 1 |  | – | – | – |
| Solid organ transplantation | 12(14.6) | 11(26.8) | 0.102 |  | – | – | – |
| Receiving corticosteroid therapy | 20(24.4) | 15(36.6) | 0.158 |  | 27(28.7) | 15(29.4) | 0.930 |
| Receiving immunosuppressive drugs | 25(30.5) | 15(36.6) | 0.496 |  | 4(4.3) | 6(11.8) | 0.165 |
| CT | | | |  |  |  |  |
| Bilateral | 77(98.7) | 41(100) | 1 |  | – | – | – |
| Node | 4(5.9) | 1(2.6) | 0.651 |  | – | – | – |
| Ground glass opacity | 70(89.7) | 36(87.8) | 0.764 |  | – | – | – |
| Thread net | 27(38) | 12(30) | 0.395 |  | – | – | – |
| Consolidation | 28(37.8) | 13(31.7) | 0.511 |  | – | – | – |
| Cavity | 2(2.9) | 0 | 0.532 |  | – | – | – |
| Pleural effusion | 19(27.1) | 8(20.5) | 0.442 |  | – | – | – |
| Lymphadenectasis | 10(14.3) | 2(5.1) | 0.206 |  | – | – | – |
| Mediastinal emphysema | 2(2.9) | 0 | 0.532 |  | – | – | – |
| Pre-ICU medication | | | | | | | |
| COVID-19 antivirals | 31(37.8) | 17(41.5) | 0.695 |  | – | – | – |
| Paxlovid | 26(31.7) | 13(31.7) | 1 |  | – | – | – |
| Bacterial antibiotic | 67(81.7) | 37(90.2) | 0.217 |  | – | – | – |
| Antifungal antibiotic | 7(8.5) | 2(4.9) | 0.716 |  | – | – | – |
| Immunoglobulin | 12(14.6) | 9(22) | 0.309 |  | – | – | – |
| Anticoagulant | 26(31.7) | 15(36.6) | 0.589 |  | – | – | – |
| Corticosteroids | 50(61) | 32(78) | 0.058 |  | – | – | – |
| Time from illness onset to ICU admission | 14(7–26) | 15(11–37.75) | 0.576 |  | 8(5.5–11.5) | 13(7–22) | 0.001 |

APACHE, acute physiology and chronic health evaluation; SOFA, sequential organ failure assessment; BMI, body mass index; ICU, intensive care unit; COVID-19, coronavirus disease 2019

Immunocompromised patients include following condition: primary immune deficiency diseases; active malignancy or malignancy within 1 y of CAP, excluding patients with localized skin cancers or early–stage cancers (eg, stage 1 lung cancer); receiving cancer chemotherapy; HIV infection with a CD4+ T lymphocyte count < 200 cells/mL or percentage < 14%; solid organ transplantation; hematopoietic stem cell transplantation; receiving corticosteroid therapy with a dose≥20 mg prednisone or equivalent daily for $ 14 d or a cumulative dose > 600 mg of prednisone; receiving biological immune modulators; receiving disease–modifying antirheumatic drugs or other immunosuppressive drugs (eg, cyclosporin, cyclophosphamide, hydroxychloroquine, methotrexate).

Supplementary, Table 5. Laboratory and vital signs of COVID-19 and influenza patients with coinfection on ICU admission

|  | COVID-19 | | |  | Influenza | | |
| --- | --- | --- | --- | --- | --- | --- | --- |
|  | None  N=82 | Coinfection  N=41 | p |  | None  N=94 | Coinfection  N=51 | p |
| Vital signs | | | | | | | |
| Temperature, ℃ | 36.8(36.5–37.48) | 37(36.6–37) | 0.784 |  | – | – | – |
| Respiratory rate, beats/min | 23(20–27) | 25(22–30) | 0.113 |  | 27(23–31) | 27(23–32) | 0.623 |
| Heart rate, beats/min | 85(72–105.5) | 99(65–110) | 0.350 |  | 86(78.75–96.25) | 88(80–101) | 0.302 |
| pH | 7.43(7.37–7.47) | 7.42(7.33–7.45) | 0.228 |  | 7.41(7.33–7.46) | 7.41(7.32–7.47) | 0.841 |
| PaCO_2_ , mmHg | 36.45(30.25–43.68) | 35(31.45–43.65) | 0.859 |  | 41(33–46) | 43.6(33–55.1) | 0.052 |
| PaO_2_/FiO_2_, mmHg | 93.85(63.5–159.73) | 96.88(70.05–156.57) | 0.304 |  | 142(102–205.63) | 160.5(115.55–207.5) | 0.310 |
| HCO_3_^–^, mmol/L | 25.4(22.5–29) | 26.25(24.05–29.38) | 0.503 |  | – | – | – |
| White blood cell, × 10^9^ /L | 8.87(6.49–12.97) | 9.47(5.59–13.95) | 0.981 |  | 7.77(4.16–12.37) | 10.25(6.17–14.03) | 0.040 |
| Neutrophil, × 10^9^ /L | 7.91(5.45–11.94) | 8.41(5.05–12.67) | 0.899 |  | – | – | – |
| Lymphocyte, × 10^9^ /L | 0.52(0.28–0.79) | 0.45(0.23–0.64) | 0.245 |  | 0.54(0.35–0.98) | 0.68(0.4–1.14) | 0.182 |
| Hemoglobin, g/L | 116(96–139) | 118(98–128) | 0.725 |  | – | – | – |
| Platelet, × 10^9^ /L | 186(132–242) | 164(117.5–204.5) | 0.141 |  | – | – | – |
| C-reactive protein, mg/L | 83.97(23.91–142.30) | 90.62(52.28–187.79) | 0.206 |  | 14.49(7.99–64.37) | 23.45(8.94–109.49) | 0.967 |
| Procalcitonin, ng/mL | 0.26(0.1–0.92) | 0.22(0.1–0.88) | 0.707 |  | 0.83(0.38–3.26) | 0.79(0.37–2.01) | 0.890 |
| Serum ferritin, μg/L | 622.65(395.05–969.43) | 732.5(579–1120.7) | 0.343 |  | – | – | – |
| Interleukin-6, pg/mL | 24.29(8.16–119.83) | 39.08(13.21–416.4) | 0.065 |  | – | – | – |
| Interleukin-8, pg/mL | 14.45(7.28–30.48) | 16.95(7.64–38.41) | 0.333 |  | – | – | – |
| CD4+ T cell | 191(132–304) | 182(74–323) | 0.266 |  | 239(139–428) | 281.5(183.75–401.25) | 0.463 |
| CD8+ T cell | 126(77–223) | 81(55–205) | 0.081 |  | 155(83–255) | 175(117–292.5) | 0.078 |
| ALT, U/L | 26(15–49) | 24(14–43.5) | 0.569 |  | 32(22.75–73.25) | 33(19–64) | 0.498 |
| TBIL, mmol/L | 1.69(7.51–15.83) | 10.18(3.94–16.16) | 0.889 |  | 11.68(7.81–19.13) | 12.48(8.22–18.6) | 0.944 |
| Cr, U/L | 82.8(56.45–125.8) | 74(57.65–145.65) | 0.903 |  | 73.65(54.3–113.25) | 61.8(47.5–94.4) | 0.052 |
| Glu, mmol/L | 10.26(7.79–14.54) | 10.83(8.53–14.41) | 0.418 |  | – | – | – |
| PT, s | 14.3(13.65–15.7) | 14.3(13.35–15.45) | 0.453 |  | 14.45(13.1–15.53) | 15(13.9–16.8) | 0.027 |
| APTT, s | 40(34.85–44.2) | 40.4(35.4–47.2) | 0.469 |  | 38.95(34.78–49.98) | 41.4(35.6–49.4) | 0.535 |
| Fibrinogen, g/L | 5.1(3.87–6.37) | 5.21(3.78–6.68) | 0.826 |  | – | – | – |
| D-dimer, μg/mL | 2.57(1.29–9.78) | 2.33(1.18–14.35) | 0.866 |  | – | – | – |

pH, power of hydrogen; PaCO_2_, partial pressure of carbon dioxide; PaO_2_/FiO_2_, partial pressure of oxygen/fraction of inspiration oxygen; ALT, alanine aminotransferase; TBIL, total bilirubin; Cr, creatinine; Glu, glucose; PT, prothrombin time; APTT, activated partial thromboplastin time

Supplementary, Table 6. Demographic and clinical Characteristics of COVID-19 and influenza patients with ICU-acquired superinfection

|  | COVID-19 N=123 | | |  | Inluenza N=145 | | |
| --- | --- | --- | --- | --- | --- | --- | --- |
|  | None  N=69 | ICU-acquired superinfection N=54 | p |  | None  N=69 | ICU-acquired superinfection  N=76 | p |
| Age (y) | 69(58–80) | 68(60–76) | 0.294 |  | 59(47–65) | 55(44–72) | 0.655 |
| Male (n%) | 54(78.3) | 45(83.3) | 0.481 |  | 44(63.8) | 51(67.1) | 0.673 |
| APECHE II | 14(11–21) | 20(14–26) | 0.004 |  | 16(13–23) | 19(14–24) | 0.202 |
| SOFA | 4(2.75–8) | 7(5–10) | 0.002 |  | 6(4–11) | 9(4.25–11) | 0.158 |
| BMI (kg/m^2^) | 24.06(20.79–25.99) | 24.05(21.45–26.81) | 0.443 |  | 24.58(22.47–27.75) | 24.7(21.77–27.04) | 0.731 |
| Smoking (n%) | 16(23.2) | 26(48.1) | 0.004 |  | 27(39.1) | 30(39.5) | 0.966 |
| Alcoholic (n%) | 13(18.8) | 15(27.8) | 0.241 |  | 18(26.1) | 23(30.7) | 0.543 |
| Vaccinated (n%) |  |  |  |  |  |  |  |
| 0 | 15(21.7) | 13(24.1) |  |  | – | – | – |
| 1 | 0 | 0 |  |  | – | – | – |
| 2 | 1(1.4) | 2(9.1) |  |  | – | – | – |
| 3 | 7(10.1) | 7(31.8) |  |  | – | – | – |
| Clinical manifestation (n%) | | | | | | | |
| Fever | 61(88.4) | 52(96.3) | 0.183 |  | 63(91.3) | 59(78.7) | 0.035 |
| Cough | 53(76.8) | 45(83.3) | 0.372 |  | 63(91.3) | 67(88.2) | 0.534 |
| Expectoration | 49(71) | 37(68.5) | 0.765 |  | 59(85.5) | 55(72.4) | 0.054 |
| Running nose | 3(4.3) | 2(3.7) | 1 |  | – | – | – |
| Rhinobyon | 1(1.4) | 1(1.9) | 1 |  | – | – | – |
| Pharyngalgia | 10(14.5) | 7(13) | 0.807 |  | – | – | – |
| Dyspnea | 58(84.1) | 54(100) | 0.002 |  | 58(84.1) | 71(93.4) | 0.072 |
| Chest stuffiness | 18(26.1) | 16(29.6) | 0.663 |  | – | – | – |
| Chest pain | 1(1.4) | 1(1.9) | 1 |  | 6(8.7) | 7(9.2) | 0.914 |
| Hemoptysis | 0 | 1(1.9) | 0.439 |  | 8(11.6) | 13(17.1) | 0.346 |
| Blood-stained sputum | 7(10.1) | 6(11.1) | 0.863 |  | – | – | – |
| Myalgias | 10(14.5) | 7(13) | 0.807 |  | – | – | – |
| Headache | 6(8.7) | 3(5.6) | 0.730 |  | – | – | – |
| Fatigue | 19(27.5) | 16(29.6) | 0.798 |  | – | – | – |
| Underlying diseases (n%) | | | | | | | |
| Hypertension | 35(50.7) | 39(72.2) | 0.016 |  | – | – | – |
| Diabetes | 24(34.8) | 25(46.3) | 0.196 |  | 23(33.3) | 26(34.2) | 0.911 |
| Chronic heart failure | 7(10.1) | 7(13) | 0.625 |  | 6(8.7) | 10(13.2) | 0.392 |
| Chronic renal failure | 15(21.7) | 12(22.2) | 0.949 |  | 8(11.6) | 5(6.7) | 0.303 |
| Chronic hepatic insufficiency | 0 | 2(3.7) | 0.191 |  | 1(1.4) | 2(2.6) | 1 |
| Chronic lung diseases | 10(14.5) | 7(13) | 0.807 |  | 17(24.6) | 16(21.1) | 0.607 |
| Connective tissue diseases | 8(11.6) | 5(9.3) | 0.676 |  | 7(10.1) | 4(5.3) | 0.268 |
| Solid malignant tumor | 4(5.8) | 4(7.4) | 0.729 |  | 6(8.7) | 4(5.3) | 0.519 |
| Hematological malignant tumor | 4(5.8) | 1(1.9) | 0.384 |  | 2(2.9) | 1(1.3) | 0.605 |
| Immunocompromised | 29(42) | 19(35.2) | 0.440 |  | – | – | – |
| Solid organ transplantation | 14(20.3) | 9(16.7) | 0.609 |  | – | – | – |
| Receiving corticosteroid therapy | 20(29) | 15(27.8) | 0.883 |  | 19(27.5) | 23(30.3) | 0.718 |
| Receiving immunosuppressive drugs | 25(36.2) | 15(27.8) | 0.321 |  | 5(7.2) | 5(6.6) | 0.565 |
| CT | | | | | | | |
| Bilateral | 65(98.5) | 53(100) | 1 |  |  | – | – |
| Node | 3(5.3) | 2(4) | 1 |  | – | – | – |
| Ground glass opacity | 56(84.8) | 50(94.3) | 0.099 |  | – | – | – |
| Thread net | 21(34.4) | 18(36) | 0.863 |  | – | – | – |
| Consolidation | 26(41.3) | 15(28.8) | 0.166 |  | – | – | – |
| Cavity | 1(1.8) | 1(2.0) | 1 |  | – | – | – |
| Pleural effusion | 13(22) | 14(28) | 0.472 |  | – | – | – |
| Lymphadenectasis | 6(10.2) | 6(12) | 0.761 |  | – | – | – |
| Mediastinal emphysema | 2(3.5) | 0 | 0.497 |  | – | – | – |
| Pre-CU medication | | | | | | | |
| COVID-19 antivirals | 29(42) | 19(35.2) | 0.440 |  | – | – | – |
| Paxlovid | 23(33.8) | 16(29.6) | 0.845 |  | – | – | – |
| Bacterial antibiotic | 52(75.4) | 52(96.3) | 0.001 |  | – | – | – |
| Antifungal antibiotic | 6(8.7) | 3(5.6) | 0.730 |  | – | – | – |
| Immunoglobulin | 14(20.3) | 7(13) | 0.284 |  | – | – | – |
| Anticoagulant | 25(36.2) | 16(29.6) | 0.441 |  | – | – | – |
| Corticosteroids | 44(63.8) | 38(70.4) | 0.441 |  | – | – | – |
| Time from illness onset to ICU admission | 18.5(8.5–29) | 14(6–17) | 0.277 |  | 9(6–15) | 10(7–16) | 0.425 |

APACHE, acute physiology and chronic health evaluation; SOFA, sequential organ failure assessment; BMI, body mass index; IPPV, intensive positive–pressure ventilation; ICU, intensive care unit

Immunocompromised patients include following condition: primary immune deficiency diseases; active malignancy or malignancy within 1 y of CAP, excluding patients with localized skin cancers or early–stage cancers (eg, stage 1 lung cancer); receiving cancer chemotherapy; HIV infection with a CD4+ T lymphocyte count < 200 cells/mL or percentage < 14%; solid organ transplantation; hematopoietic stem cell transplantation; receiving corticosteroid therapy with a dose≥20 mg prednisone or equivalent daily for ≥14 d or a cumulative dose > 600 mg of prednisone; receiving biological immune modulators; receiving disease–modifying antirheumatic drugs or other immunosuppressive drugs (eg, cyclosporin, cyclophosphamide, hydroxychloroquine, methotrexate).

Supplementary, Table 7. Laboratory and vital signs of COVID-19 and influenza patients with ICU-acquired superinfection on ICU admission

|  | COVID-19 | | |  | Influenza | | |
| --- | --- | --- | --- | --- | --- | --- | --- |
|  | None  N=69 | ICU-acquired superinfection  N=54 | p |  | None  N=69 | ICU-acquired superinfection  N=76 | p |
| Vital signs | | | | | | | |
| Temperature, ℃ | 36.8(36.5–37) | 37(36.5–37.8) | 0.188 |  | – | – | – |
| Respiratory rate, beats/min | 23(20–27) | 25(21.25–30) | 0.113 |  | 27(23–30.5) | 27(24–32) | 0.537 |
| Heart rate, beats/min | 85(71.25–105) | 94(68–110) | 0.307 |  | 86(80–97) | 87.5(78.25–100.5) | 0.791 |
| pH | 7.44(7.39–7.48) | 7.40(7.33–7.45) | 0.002 |  | 7.43(7.34–7.46) | 7.39(7.30–7.46) | 0.316 |
| PaCO_2_ , mmHg | 34.65(30.58–40.3) | 38(29.8–47.08) | 0.079 |  | 38.55(32.73–49) | 43.5(34.08–49.63) | 0.174 |
| PaO_2_/FiO_2_, mmHg | 111.67(61.25–189.33) | 85.8(69.7–127.4) | 0.224 |  | 150.6(105.75–214) | 144(101–200) | 0.539 |
| HCO_3_^–^, mmol/L | 25.3(22.45–27.5) | 27.1(24.1–31.2) | 0.219 |  | – | – | – |
| White blood cell, × 10^9^ /L | 8.69(5.95–11.79) | 10.5(7.05–15.43) | 0.034 |  | 7.96(4.31–11.3) | 10.94(4.46–14.45) | 0.054 |
| Neutrophil, × 10^9^ /L | 7.31(5.03–10.52) | 9.34(6–14.46) | 0.018 |  | – | – | – |
| Lymphocyte, × 10^9^ /L | 0.55(0.29–0.80) | 0.45(0.21–0.61) | 0.046 |  | 0.63(0.34–1.07) | 0.61(0.37–0.93) | 0.831 |
| Hemoglobin, g/L | 117(95.75–131) | 117.5(96.75–143) | 0.394 |  | – | – | – |
| Platelet, × 10^9^ /L | 174(124.5–226.25) | 178(121–236.75) | 0.740 |  | – | – | – |
| C-reactive protein, mg/L | 59.76(36.96–117.90) | 118.8(62.63–195.7) | 0.005 |  | 41.7(8.99–134.97) | 32.8(7.99–127.17) | 0.182 |
| Procalcitonin, ng/mL | 0.22(0.1–0.61) | 0.32(0.13–1.36) | 0.041 |  | 0.81(0.32–1.93) | 0.85(0.41–4.01) | 0.290 |
| Serum ferritin, μg/L | 639.2(507.85–904.45) | 734.25(380.68–1253.48) | 0.899 |  | – | – | – |
| Interleukin-6, pg/mL | 17.91(4.53–127.06) | 63.74(15.77–258.58) | 0.006 |  | – | – | – |
| Interleukin-8, pg/mL | 12.59(5.24–24.21) | 20.67(10.48–44.05) | 0.005 |  | –– | – | – |
| CD4+ T cell | 217(123.75–403.25) | 180(96.25–274.75) | 0.117 |  | 250(159.75–435) | 262(133–391) | 0.562 |
| CD8+ T cell | 141.5(86–296.75) | 85(55.5–169.75) | 0.004 |  | 160(101–313) | 165(91.75–256.25) | 0.603 |
| ALT, U/L | 25(15–47.75) | 26(15–43.75) | 0.928 |  | 34(21–72.5) | 30(20.25–74.5) | 0.406 |
| TBIL, mmol/L | 11.96(7.75–15.74) | 8.85(6.83–17.47) | 0.274 |  | 11.51(8.59–17.2) | 12.29(7.63–22.59) | 0.815 |
| Cr, U/L | 73.7(56–125.78) | 83.85(59.08–162.55) | 0.405 |  | 65.2(52.75–104.25) | 69.9(49.63–111.63) | 0.866 |
| Glu, mmol/L | 9.78(7.5–12.6) | 12.17(9–16.55) | 0.005 |  | – | – | – |
| PT, s | 14.15(13.43–14.93) | 14.45(13.6–15.75) | 0.462 |  | 14.3(13.25–15.35) | 14.85(14–16.73) | 0.28 |
| APTT, s | 39.9(35.45–45.3) | 40.35(34.85–45.78) | 0.994 |  | 41.75(38–48.93) | 40.1(35.4–52.2) | 0.459 |
| Fibrinogen, g/L | 5.07(3.78–6.36) | 5.29(4.15–6.64) | 0.516 |  | – | – | – |
| D-dimer, μg/mL | 2.31(1.11–5.86) | 4.14(1.52–18.92) | 0.026 |  | – | – | – |

pH, power of hydrogen; PaCO_2_, partial pressure of carbon dioxide; PaO_2_/FiO_2_, partial pressure of oxygen/fraction of inspiration oxygen; ALT, alanine aminotransferase; TBIL, total bilirubin; Cr, creatinine; Glu, glucose; PT, prothrombin time; APTT, activated partial thromboplastin time

Supplementary, Table 8. Comparison of demographic and clinical characteristics between survivor and non-urvival of COVID-19 and patients

|  | COVID-19 N=123 | | |  | Influenza N=145 | | |
| --- | --- | --- | --- | --- | --- | --- | --- |
|  | Non-survivor  N=68 | Survivor  N=55 | p |  | Non-survivor  N=68 | Survivor  N=77 | p |
| Age (y) | 69(60–79) | 67(56–78) | 0.495 |  | 61(48–71) | 56(43–67) | 0.157 |
| Male (n%) | 54(79.4） | 45(81.8) | 0.738 |  | 50(73.5) | 45(58.4) | 0.056 |
| APECHE II | 19(15–26) | 13(10–19) | <0.001 |  | 21(14–27) | 16(11–20) | <0.001 |
| SOFA | 8(5–11) | 4(2–6) | <0.001 |  | 9(6–12) | 6(3–10) | 0.001 |
| BMI (kg/m^2^) | 24.11(22.04–26.89) | 23.66(20.50–25.39) | 0.178 |  | 23.9(22.1–26.3) | 25.3(22.1–28.7) | 0.045 |
| Smoking (n%) | 26(38.2) | 16(29.1) | 0.288 |  | 30(44.1) | 27(35.1) | 0.265 |
| Alcoholic (n%) | 16(23.5) | 12(21.8) | 0.822 |  | 20(29.4) | 21(27.6) | 0.813 |
| Vaccinated (n%) | | | | | | | |
| 0 | 20(69) | 8(50.0) |  |  | – | – | – |
| 1 |  |  |  |  | – | – | – |
| 2 | 2(6.9) | 1(6.3) |  |  | – | – | – |
| 3 | 7(24.1) | 7(43.8) |  |  | – | – | – |
| Clinical manifestation (n%) | | | | | | | |
| Fever | 61(89.7) | 52(94.5) | 0.509 |  | 59(86.8) | 63(82.9) | 0.519 |
| Cough | 55(80.9) | 43(78.2） | 0.711 |  | 62(91.2) | 68(88.3) | 0.572 |
| Expectoration | 45(66.2) | 41(74.5) | 0.314 |  | 56(82.4) | 58(75.3) | 0.303 |
| Running nose | 2(2.) | 3(5.5) | 0.656 |  | – | – | – |
| Rhinobyon | 1(1.5) | 1(1.8) | 1 |  | – | – | – |
| Pharyngalgia | 10(14.7) | 7(12.7) | 0.752 |  | – | – | – |
| Dyspnea | 67(98.5) | 45(81.8) | 0.001 |  | 64(94.1) | 65(84.4) | 0.063 |
| Chest stuffiness | 16(23.5) | 18(32.7) | 0.257 |  |  |  |  |
| Chest pain | 2(2.9) | 0 | 0.502 |  | 9(13.2) | 4(5.2) | 0.091 |
| Hemoptysis | 1(1.5) | 0 | 1 |  | 13(19.1) | 8(10.4) | 0.136 |
| Blood–stained sputum | 8(11.8) | 5(9.1) | 0.771 |  | – | – | – |
| Myalgias | 9(13.2) | 8(14.5) | 0.834 |  | – | – | – |
| Headache | 3(4.4) | 6(10.9) | 0.296 |  | – | – | – |
| Fatigue | 19(27.9) | 16(29.1) | 0.888 |  | – | – | – |
| Hypertension | 46(67.6) | 28(50.9) | 0.059 |  | – | – | – |
| Diabetes | 31(45.6) | 18(32.7) | 0.147 |  | 26(38.2) | 23(29.9) | 0.288 |
| Chronic heart failure | 11(16.2) | 3(5.5) | 0.063 |  | 7(10.3) | 9(11.7) | 0.789 |
| Chronic renal failure | 14(20.6) | 13(23.6) | 0.685 |  | 7(10.3) | 6(7.9) | 0.616 |
| Chronic hepatic insufficiency | 1(1.5) | 1(1.8) | 1 |  | 2(2.9) | 1(1.3) | 0.600 |
| Chronic lung diseases | 11(16.2) | 6(10.9) | 0.400 |  | 21(30.9) | 12(15.6) | 0.028 |
| Connective tissue diseases | 8(11.8) | 5(9.1) | 0.632 |  | 8(11.8) | 3(3.9) | 0.074 |
| Solid malignant tumor | 5(7.4) | 3(5.5) | 0.730 |  | 5(7.4) | 5(6.5) | 1 |
| Hematological malignant tumor | 2(2.9) | 3(5.5) | 0.656 |  | 2(2.9) | 1(1.3) | 0.600 |
| Immunocompromised | 28(41.2) | 20(36.4) | 0.586 |  | – | – | – |
| Solid organ transplantation | 12(17.6) | 11(20) | 0.739 |  | – | – | – |
| Receiving corticosteroid therapy | 21(30.9) | 14(25.5) | 0.507 |  | 27(39.7) | 15(19.5) | 0.007 |
| Receiving immunosuppressant | 22(32.4) | 18(32.7) | 0.965 |  | 4(5.9) | 6(7.8) | 0.750 |
| CT | | | | | | | |
| Bilateral | 66(100) | 52(98.1) | 0.445 |  | – | – | – |
| Node | 2(3.2) | 3(6.7) | 0.648 |  | – | – | – |
| Ground glass opacity | 60(90.9) | 46(86.8) | 0.474 |  | – | – | – |
| Thread net | 24(38.7) | 15(30.6) | 0.375 |  | – | – | – |
| Consolidation | 22(34.4) | 19(37.3) | 0.749 |  | – | – | – |
| Cavity | 2(3.2) | 0 | 0.508 |  | – | – | – |
| Pleural effusion | 18(29) | 9(19.1) | 0.236 |  | – | – | – |
| Lymphadenectasis | 6(9.7) | 6(12.8) | 0.610 |  | – | – | – |
| Mediastinal emphysema | 0 | 2(4.4) | 0.175 |  | – | – | – |
| Pre-ICU medication | | | | | | | |
| COVID-19 antivirals | 27(39.7) | 21(38.2) | 0.863 |  | – | – | – |
| Paxlovid | 21(30.9) | 18(32.7) | 0.231 |  | – | – | – |
| Bacterial antibiotic | 57(83.8) | 47(85.5) | 0.803 |  | – | – | – |
| Antifungal antibiotic | 6(8.8) | 3(5.5) | 0.730 |  | – | – | – |
| Immunoglobulin | 11(16.2) | 10(18.2) | 0.769 |  | – | – | – |
| Anticoagulant | 18(26.5) | 23(41.8) | 0.073 |  | – | – | – |
| Corticosteroids | 39(57.4) | 43(78.2) | 0.015 |  | – | – | – |
| Time from illness onset to ICU admission | 11.5(7.25–17) | 14(11–24) | 0.006 |  | 11(7–20.75) | 7.5(6–10.75) | <0.001 |

APACHE, acute physiology and chronic health evaluation; SOFA, sequential organ failure assessment; BMI, body mass index; IPPV, intensive positive–pressure ventilation; ICU, intensive care unit

Immunocompromised patients include following condition: primary immune deficiency diseases; active malignancy or malignancy within 1 y of CAP, excluding patients with localized skin cancers or early–stage cancers (eg, stage 1 lung cancer); receiving cancer chemotherapy; HIV infection with a CD4+ T lymphocyte count < 200 cells/mL or percentage < 14%; solid organ transplantation; hematopoietic stem cell transplantation; receiving corticosteroid therapy with a dose≥20 mg prednisone or equivalent daily for ≥14 d or a cumulative dose > 600 mg of prednisone; receiving biological immune modulators; receiving disease–modifying antirheumatic drugs or other immunosuppressive drugs (eg, cyclosporin, cyclophosphamide, hydroxychloroquine, methotrexate).

Supplementary, Table 9. Comparison of laboratory and vital signs between survivor and non–survival of COVID-19 patients

|  | COVID-19 N=123 | | |  | Influenza N=145 | | |
| --- | --- | --- | --- | --- | --- | --- | --- |
|  | Non-survivor  N=68 | Survivor  N=55 | p |  | Non-survivor  N=68 | Survivor  N=77 | p |
| Vital signs |  |  |  |  |  |  |  |
| Temperature, ℃ | 37(36.5–37.8) | 36.8(36.5–37.2) | 0.399 |  | – | – | – |
| Respiratory rate, beats/min | 24(20–28) | 24(20–28) | 0.550 |  | 28(25–33) | 26(22–30) | 0.028 |
| Heart rate, beats/min | 95(77–110) | 80(67–100) | 0.021 |  | 88(79–104) | 85(77–95) | 0.157 |
| pH | 7.41(7.33–7.45) | 7.44(7.41–7.48) | 0.004 |  | 7.40(7.33–7.46) | 7.42(7.33–7.46) | 0.677 |
| PaCO_2_ , mmHg | 35.8(28.9–44) | 36.2(31.6–43.6) | 0.580 |  | 43.3(33.18–54.6) | 41.75(33–47) | 0.248 |
| PaO_2_/FiO_2_, mmHg | 83.5(63.65–136.77) | 111.25(72.44–181.09) | 0.058 |  | 127(92–189) | 174(132–229) | 0.001 |
| HCO_3_^–^, mmol/L | 25.4(22.75–29.85) | 25.35(22.53–28.40) | 0.776 |  | – | – | – |
| White blood cell, × 10^9^ /L | 10.67(8.17–14.01) | 7.72(5.68–11.75) | 0.021 |  | 10.31(6.63–14.16) | 6.85(4.03–11.88) | 0.004 |
| Neutrophil, × 10^9^ /L | 8.99(6.77–12.85) | 6.63(4.88–10.41) | 0.020 |  | – | – | – |
| Lymphocyte, × 10^9^ /L | 0.47(0.24–0.64) | 0.53(0.29–0.80) | 0.135 |  | 0.6(0.34–1.04) | 0.68(0.37–1.02) | 0.551 |
| Hemoglobin, g/L | 118(96–144) | 116(98–130) | 0.159 |  | – | – | – |
| Platelet, × 10^9^ /L | 168(118–235) | 190(129–230) | 0.317 |  | – | – | – |
| C-reactive protein, mg/L | 97.22(48.48–190.8) | 82.38(36.39–132.41) | 0.071 |  | 12(7.42–48.32) | 24.73(10.50–107.21) | 0.438 |
| Procalcitonin, ng/mL | 0.41(0.14–1.3) | 0.21(0.1–0.43) | 0.004 |  | 0.83(0.4–2.12) | 0.82(0.33–3.30) | 0.987 |
| Serum ferritin, μg/L | 709.15(417.53–1240.45) | 646.3(403.3–890.2) | 0.377 |  | – | – | – |
| Interleukin-6, pg/mL | 77.11(18.10–302) | 13(4.26–62.41) | <0.001 |  | – | – | – |
| Interleukin-8, pg/mL | 19.37(10.38–46) | 11.34(5.43–20.86) | 0.001 |  | – | – | – |
| CD4+ T cell | 187(97.5–286) | 206(123.5–433.5) | 0.137 |  | 209(111–332) | 319(199–456) | 0.011 |
| CD8+ T cell | 89(56–194) | 129(82–313.5) | 0.028 |  | 140(79–266) | 178(108–274) | 0.151 |
| ALT, U/L | 23(14–35) | 33(17–51) | 0.029 |  | 32(23–54.5) | 32(20–95) | 0.846 |
| TBIL, mmol/L | 11.18(7.68–17.23) | 9.87(7.05–14.25) | 0.485 |  | 11.68(9.04–20.38) | 11.92(7.44–17.86) | 0.273 |
| Cr, U/L | 90.7(59.4–171.9) | 69.4(56–109.4) | 0.007 |  | 69.85(55.3–101) | 65.2(48.6–113.35) | 0.576 |
| Glu, mmol/L | 11.4(8.61–16.55) | 9.61(7.77–12.54) | 0.038 |  | – | – | – |
| PT, s | 14.4(13.7–15.9) | 14.1(13.4–14.7) | 0.101 |  | 14.9(14.1–16.8) | 14.3(13.1–15.35) | 0.007 |
| APTT, s | 40.3(35.3–47.6) | 40(35.4–43.4) | 0.400 |  | 41.65(36.25–52.3) | 37.8(35.5–41.3) | 0.123 |
| Fibrinogen, g/L | 5.04(3.86–6.55) | 5.35(3.87–6.45) | 0.879 |  | – | – | – |
| D-dimer, μg/mL | 4.1(1.93–18.87) | 1.81(0.81–5.86) | <0.001 |  | – | – | – |

pH, power of hydrogen; PaCO_2_, partial pressure of carbon dioxide; PaO_2_/FiO_2_, partial pressure of oxygen/fraction of inspiration oxygen; ALT, alanine aminotransferase; TBIL, total bilirubin; Cr, creatinine; Glu, glucose; PT, prothrombin time; APTT, activated partial thromboplastin time

Supplementary, Table 10. Comparison of treatment and outcomes between survivor and non-survival of COVID-19 and influenza patients

|  | COVID-19 N=123 | | |  | Influenza N=145 | | |
| --- | --- | --- | --- | --- | --- | --- | --- |
|  | Non-survivor  N=68 | Survivor  N=55 | p |  | Non-survivor  N=68 | Survivor  N=77 | p |
| Paxlovid | 42(63.6) | 25(49) | 0.113 |  | – | – | – |
| Corticosteroids | 60(90.9) | 43(84.3) | 0.276 |  | – | – | – |
| Tocilizumab | 14(21.2) | 6(11.8) | 0.178 |  | – | – | – |
| Baricitinib | 10(15.2) | 8(15.7) | 0.937 |  | – | – | – |
| Tofacitinib | 3(4.5) | 0 | 0.256 |  | – | – | – |
| Anticoagulation | 62(93.9) | 49(96.1) | 0.695 |  | – | – | – |
| Bacterial antibiotic | 63(95.5) | 48(94.1) | 1 |  | – | – | – |
| Antifungal antibiotic | 37(56.1) | 19(38) | 0.054 |  | – | – | – |
| Barotrauma | 8(13.6) | 5(12.8) | 0.916 |  | 4(5.9) | 4(5.2) | 1 |
| Acute kidney injury, n% | 28(43.8) | 5(11.1) | <0.001 |  | 43(63.2) | 19(24.7) | <0.001 |
| Cardiovascular failure, n% | 38(59.4) | 4(8.9) | <0.001 |  | 28(41.2) | 7(9.1) | <0.001 |
| Acute liver injury, n% | 10(15.6) | 3(6.7) | 0.155 |  | 15(22.1) | 4(5.2) | 0.003 |
| Hospital acquired pneumonia, n% | 40(58.8) | 14(25.5) | <0.001 |  | 35(51.5) | 41(53.2) | 0.831 |
| Urinary infection, n% | 2(3.1) | 2(4.2) | 1 |  | 0 | 2(2.6) | 0.498 |
| Abdominal infection, n% | 2(3.1) | 1(2.2) | 1 |  | 2(2.9) | 0 | 0.218 |
| Bloodstream infection, n% | 6(9.2) | 1(2.2) | 0.236 |  | 3(4.4) | 1(1.3) | 0.341 |
| Deep venous thrombosis, n% | 13(21) | 15（31.9） | 0.195 |  | – | – | – |
| Pulmonary embolism, n% | 1(1.6) | 1(2.2) | 1 |  | – | – | – |
| Gastrointestinal bleeding, n% | 18(29) | 8(17) | 0.145 |  | – | – | – |
| The need of CRRT, n% | 27(43.5) | 4(8.90) | <0.001 |  | 31(45.6) | 19(24.7) | 0.008 |
| The need of tracheal intubation, n% | 51(76.1) | 18(32.7) | <0.001 |  | 63(92.6) | 42(55.3) | <0.001 |
| The need of tracheotomy, n% | 28(41.8) | 14(25.9) | 0.068 |  | – | – | – |
| The need of ECMO, n% | 8(12.3) | 3(6.3) | 0.349 |  | 22(32.4) | 14(18.2) | 0.049 |
| The need of prone position, n% | 28(43.8) | 15(30.6) | 0.154 |  | – | – | – |
| The need of recruitment, n% | 7(10.8) | 0 | 0.040 |  | – |  | – |
| Coinfection | 26(38.2) | 15(27.3) | 0.200 |  | 28(41.2) | 23(29.9) | 0.155 |
| ICU acquired superinfection | 40(58.8) | 14(25.5) | <0.001 |  | 35(51.5) | 41(53.2) | 0.831 |
| The length of IPPV, days | 6(1–12) | 0(0–5) | <0.001 |  | 7(5–13.75) | 15.5(6–39.5) | 0.004 |
| ICU length of stay, days | 9.5(6–15) | 7.5(3–13.25) | 0.073 |  | 10(6–18) | 11.5(6–34.75) | 0.151 |
| Hospital length of stay, days | 9(6–15) | 17(12–25) | <0.001 |  | 10(6–17.75) | 18(8.25–10.75) | 0.001 |

CRRT, continuous renal replacement therapy; ECMO, extracorporeal membrane oxygenation; IPPV, intensive positive–pressure ventilation; ICU, intensive care unit
